# Noncanonical amino acids as prophage inducers for protein regulation in bacteria-based delivery systems

**DOI:** 10.1128/mbio.03988-24

**Published:** 2025-03-14

**Authors:** Hongfang Liu, Sijia Shen, Qi Xu, Yuyang Wang, Kejing Qi, Bowen Lu, Bing Tang, Min Wu, Fei Gan

**Affiliations:** 1 State Key Laboratory of Metabolism and Regulation in Complex Organisms, Hubei Key Laboratory of Cell Homeostasis, College of Life Science, TaiKang Center for Life and Medical Sciences, Wuhan University, Wuhan, China; 2 State Key Laboratory of Metabolism and Regulation in Complex Organisms, Frontier Science Center for Immunology and Metabolism, Hubei Key Laboratory of Cell Homeostasis, Hubei Key Laboratory of Developmentally Originated Disease, College of Life Sciences, TaiKang Center for Life and Medical Sciences, Renmin Hospital of Wuhan University, Wuhan University, Wuhan, China; 3 Hubei Key Laboratory of Cell Homeostasis, State Key Laboratory of Virology, College of Life Sciences, Wuhan University, Wuhan, China; Korea Advanced Institute of Science and Technology, Daejeon, South Korea

**Keywords:** genetic code expansion, noncanonical amino acid, λ phage, bacteria-based delivery system

## Abstract

Genetically engineered bacteria represent a promising drug delivery tool for disease treatment. The development of new strategies for specific and independent protein regulation is necessary, especially for combination protein drug therapy. Using the well-studied *Escherichia coli* phage λ as a model system, we applied noncanonical amino acids (ncAAs) as novel inducers for protein regulation in a bacteria-based delivery system. Screening the permissive sites of the Cro protein revealed that incorporation of AlocK at the K8 site with the *Mb*PylRS-349F/tRNA^Pyl^ system produced a functional Cro-K8AlocK variant. Using an engineered λ lysogen expressing the *Mb*PylRS-349F/tRNA^Pyl^ pair, Cro-8X, and the reporter mNeonGreen, *in vitro* and *in vivo* experiments showed that AlocK led to bacterial lysis through prophage activation and the release of mNeonGreen. If *mNeonGreen* was integrated into the λ prophage genome, λ phages released due to AlocK induction delivered the reporter gene into the recipient *E. coli* strain, enabling mNeonGreen expression. Furthermore, insertion of pIF at the F14 site with the *Af*pIFRS/tRNA^Tyr^ pair produced a functional Cro-F14pIF variant. Importantly, *Af*pIFRS/tRNA^Tyr^ and *Mb*PylRS-349F/tRNA^Pyl^ pairs were confirmed to be mutually orthogonal. In a mixture of two engineered λ lysogens expressing different aaRS/tRNAs, Cro-ncAAs, and reporter proteins, AlocK and pIF independently induced bacterial lysis and activated the expression of mNeonGreen and mCherry in the recipient *E. coli* strain. Collectively, the proposed bacteria-based delivery system provides two options for protein delivery and enables independent regulation of multiple proteins with ncAAs, offering a novel approach for *in situ* protein regulation and combination therapy.

**IMPORTANCE:**

The use of genetically engineered bacteria as drug delivery vectors has attracted more and more attention in recent years. A key issue with bacteria-based delivery systems is how to regulate multiple protein drugs. Based on genetic code expansion technology, we developed a new strategy of using ncAAs as small molecular inducers for *in situ* protein regulation and engineered λ phage lysogen into a bacteria-based delivery system that can function in two delivery modes. Furthermore, this strategy enables independent regulation of multiple proteins by different ncAAs, offering important implications for combination therapy. This approach requires minimal genetic engineering efforts, and similar strategies can be applied to engineer other prophage-bacteria systems or study phage biology. This work expands the therapeutic applications of ncAAs and lysogenic phages.

## INTRODUCTION

Advances in synthetic biology have been contributing to diverse research areas, from basic biology to biomanufacturing and disease therapy. Inspired by the idea of designing and assembling bio-parts or bio-components to understand and manipulate living systems, engineering bacteria as drug delivery systems is under rapid development, which exhibits great potential to offer targeted, efficient, and cost-effective treatments, particularly in the context of cancer therapy (1). Due to its tumor tropism, *Salmonella enterica* serovar *Typhimurium* strain has been engineered into an autonomously lysing bacterial system to invade and deliver protein drugs into tumor cells for treatment (2) and also been engineered with quorum sensing-related elements to achieve periodic bacterial cell lysis for controlled release of target proteins in tumor tissues (3). Additionally, the probiotics *Escherichia coli* Nissle 1917 were engineered to produce and release the AvCystatin protein to treat inflammatory bowel diseases (4). In these engineered bacteria, the regulated expression and controlled release of protein drugs play a crucial role (5). Current bacteria-based delivery systems mostly focus on producing a single therapeutic protein, which may potentially result in drug resistance and reduced efficacy over long-term usage. While treatment with multiple protein drugs confers higher efficacy, expression of all the proteins in the same bacterial chassis can confer metabolic burdens for the cells. Alternatively, the protein drugs can be produced individually by an engineered strain, which is mixed to formulate strain cocktails for combination therapy. Studies have reported approaches using quorum lysis and constitutive or inducible promoters for protein regulation purposes; however, in those cases, the release or production of protein drugs was controlled by the same factor (3, 6, 7). Besides isopropyl β-D-1-thiogalactopyranoside and tetracycline (8, 9), the repertoire of small molecules available for protein regulation is limited. The development of new strategies to regulate protein drugs specifically and independently is necessary, especially for combination protein drug therapy.

Noncanonical amino acids (ncAAs) that contain customized side groups other than the conventional 20 amino acids are excellent candidates for use as small molecular inducers. Genetic code expansion technology enables the site-specific incorporation of a given ncAA into proteins in response to unique codons (typically the amber nonsense codon) by introducing orthogonal aminoacyl-tRNA synthetase (aaRS)/tRNA pairs into cells (10–12). To date, aaRS/tRNA pairs from various species have been reported, including the TyrRS/tRNA^Tyr^ pair from *Methanococcus jannaschii* (*Mj*TyrRS/tRNA^Tyr^) and the PylRS/tRNA^Pyl^ pair from *Methanosarcina barkeri* (*Mb*PylRS/tRNA^Pyl^) or *Methanosarcina mazei* (*Mm*PylRS/tRNA^Pyl^) (13–16). More than 300 ncAAs that are chemically synthesized with novel properties have been incorporated into proteins in living systems, including virus, bacteria, yeast, and mammalian cells (17–19). By introducing a stop codon into the target gene, ncAAs can also serve as switches to regulate gene expression in cells engineered to express an appropriate aaRS/tRNA pair. Using this strategy, an OmeY-triggered therapeutic switch system composed of a bacterial aaRS/tRNA pair and an insulin gene carrying an amber codon was engineered into mammalian cells to achieve blood glucose control in diabetic model mice (20). Moreover, when the stop codon is introduced into an essential gene, the functional full-length protein can only be produced with ncAA incorporation, restraining the strict growth dependence of engineered cells on ncAAs and conferring strong resistance to horizontal gene transfer (21–24). This strategy has been applied to engineer microbes as conditional vaccines for both viral and bacterial infections (25, 26), showcasing its effectiveness in biocontainment.

**Fig 1:**
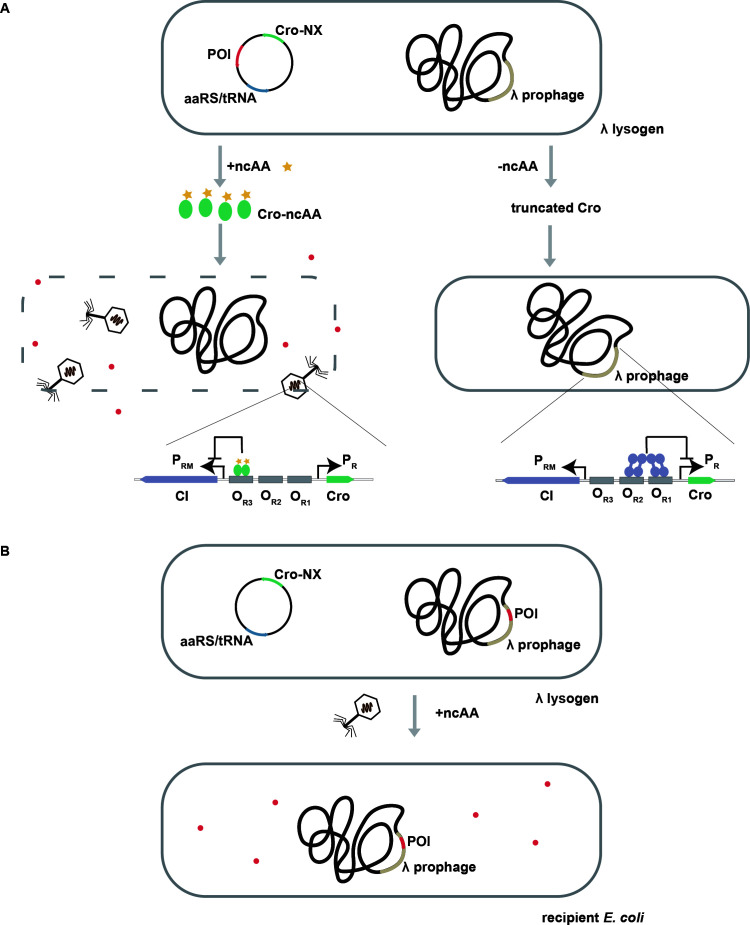
Diagram illustrating bacteria-based delivery with ncAAs as inducers. (A) Protein release mediated by λ prophage induction with ncAAs (Mode 1). In the λ lysogen engineered to express an aaRS/tRNA pair, Cro-ncAA variant, and proteins of interest (POI), in the presence of ncAAs (yellow pentagon), the Cro-ncAA variant (green oval) triggers the λ phage to enter the lytic cycle, leading to bacterial cell lysis and release of POI (red dot). In the absence of ncAAs, the prophage maintains the lysogenic status due to the inhibition of the P_R_ promoter by CI protein (purple oval). (B) Sustained protein expression mediated by ncAAs (Mode 2). When the *POI* gene is inserted into the λ prophage genome, the released λ phage progenies further deliver the *POI* gene into a provided *E. coli* recipient strain, activating POI expression.

Based on genetic code expansion, we propose a strategy of using ncAAs as inducers to regulate proteins of interest (POIs) via prophage activation in bacteria-based delivery systems and used the well-studied *E. coli* phage λ as proof of concept in our study (Fig. 1). This strategy relies on the regulation of the λ phage life cycle, which is controlled mainly by phage-encoded repressor CI and the anti-repressor Cro (27, 28). The *cI* and *cro* genes are located adjacently on the genome and share the operator sequence O_R_ composed of O_R1_, O_R2_, and O_R3_ (29, 30). Following target bacterial host infection, the λ phage can integrate its genome into the bacterial genome and enter the lysogenic cycle. The CI protein binds to the O_R1_ and O_R2_ sites and represses the expression of *cro* and other genes, stabilizing the lysogenic status (29). Upon external signal stimulation, such as ultraviolet light-induced DNA damage, the SOS response is activated, and RecA induces the cleavage of the CI protein, causing the λ phage to enter the lytic cycle with progeny phages released upon host cell lysis (31). Cro can bind to the O_R3_ site, repressing *cI* gene expression and maintaining the lytic status (32). In this work, we plan to control the expression of Cro protein with ncAAs and engineer λ lysogen into a delivery system (Fig. 1). In the absence of ncAAs, expression of the Cro terminates at the in-frame TAG codon to give a truncated non-functional peptide, and the λ phage remains lysogenic. Incorporation of a ncAA at the TAG site results in the full-length ncAA-containing Cro (Cro-ncAA) variant, triggering the lysogenic to lytic transition of the λ phage lifecycle and, consequently, to cell lysis. When a POI is expressed in the λ lysogen, it will be released upon lysis in the presence of ncAAs, resulting in a one-shot release of the POI (Mode 1, Fig. 1A). When the *POI* gene is integrated into the λ phage genome, POI expression is repressed in the λ lysogen. Upon ncAA addition, the λ phage progenies are released and deliver the *POI* gene into the supplied recipient *E. coli* strain where POI expression is activated (Mode 2, Fig. 1B). This strategy provides two options for protein delivery and should also enable independent regulation of multiple proteins with different ncAAs.

## RESULTS

### Incorporation of ncAA into Cro with efficiency and functionality

**Fig 2:**
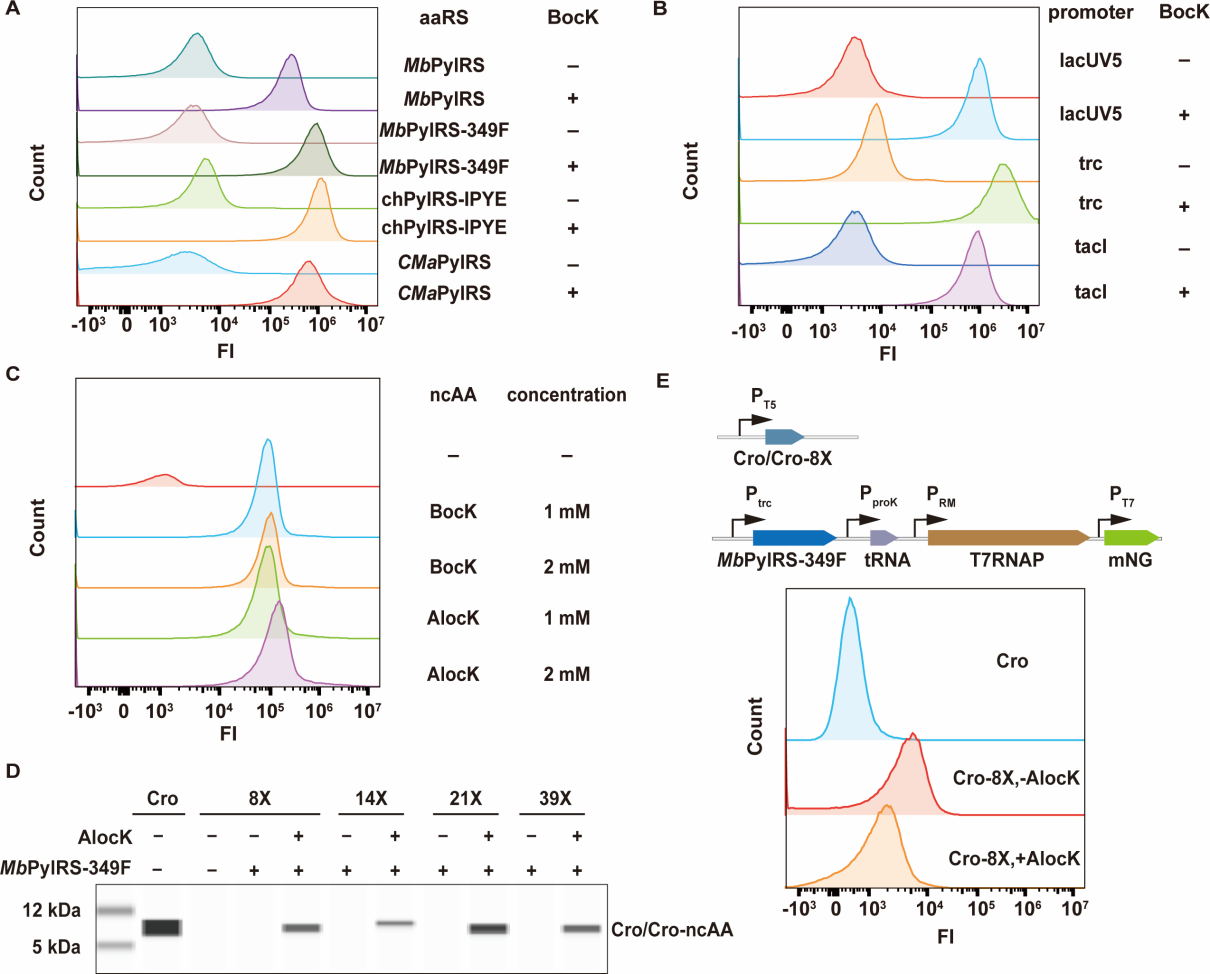
Incorporation of ncAA in Cro and function assay of Cro-ncAA variants. (A) Incorporation of ncAA (1 mM BocK) with *Mb*PylRS, *Mb*PylRS-349F, *CMa*PylRS, and chPylRS-IPYE that was expressed with tacI promoter. (B) Incorporation of ncAA (1 mM BocK) with *Mb*PylRS-349F that was expressed with different promoters. (C) Incorporation of different ncAAs (BocK and AlocK) with *Mb*PylRS-349F. (D) Simple western immunoblotting to detect the incorporation of AlocK at selected sites of Cro protein. The molecular weight of Cro is 7.4 kDa. (E) Cro-ncAA function assay. The top and bottom panels present the construct design and the mNG fluorescent signals measured with flow cytometry, respectively. Functional wild-type Cro (Cro) and variant Cro-K8AlocK (Cro-8X, +AlocK) bind to the P_RM_ promoter and inhibit mNG expression. FI: fluorescence intensity. mNG: mNeonGreen. “X”: an amber codon introduced at the site to encode ncAA.

Efficient ncAA incorporation to produce active Cro-ncAA variants is essential for ensuring proper function and control of the bacteria-based delivery system. Because the orthogonal aaRS and tRNA, the key components of genetic code expansion, are important for efficient ncAA incorporation (14), we first evaluated several aaRS/tRNA pairs and their cognate ncAAs that have been individually reported to exhibit high efficiency in parallel (Fig. S1 and S2). These orthogonal aaRSs include a mutant of *M. barkeri-*derived pyrrolysyl-tRNA synthetase (*Mb*PylRS-349F) (33), a chimeric mutant of PylRSs from *M. barkeri* and *M. mazei* (chPylRS-IPYE) (34), a PylRS derived from *Candidatus Methanomethylophilus alvus* (*CMa*PylRS) (35) (Fig. 2A; Fig. S3A), and mutants of tyrosyl-tRNA synthetase from *Archaeoglobus fulgidus* (*Af*pIFRS) (36) and *M. jannaschii* (pAzFRS) (37) (Fig. S4). The fluorescent protein mNeonGreen containing the TAG codon at position 42 (mNG-42X) or super-folder GFP containing the TAG codon at position 39 (sfGFP-39X) was used as the reporter. In the absence of ncAAs, translation is terminated at the TAG codon, resulting in a truncated reporter protein. When supplemented with ncAAs, insertion of a ncAA in response to the TAG codon leads to the synthesis of the full-length fluorescent reporter protein, and the ncAA incorporation efficiency is reflected by the fluorescence intensity. Plasmids for expression of the aaRS/tRNA pairs and the reporter protein were transformed into *E. coli* DH10B, and flow cytometry analysis was applied to measure the fluorescence of the resulting recombinant strains. Each point on the flow cytometry curve represents the number of bacterial cells (*y* axis) exhibiting a specific fluorescence intensity (*x* axis). A shift of the peak toward the right indicates an increase in fluorescence intensity, signifying a higher proportion of cells expressing a full-length fluorescent reporter protein. The median fluorescence intensity values of samples were used to facilitate comparisons (Fig. S3). Flow cytometry analysis results showed that *Mb*PylRS-349F and chPylRS-IPYE exhibited the highest ncAA incorporation activities (Fig. 2A; Fig. S3A and S4). Because unintended full-length proteins may be produced by natural amino acid incorporation, which is primarily determined by the properties of the aaRSs, we selected *Mb*PylRS-349F for further studies due to its minimal incorporation of natural amino acids. When *Mb*PylRS-349F was expressed individually with the promoter lacUV5, tacI, or trc, expression with the trc promoter yielded the highest ncAA incorporation efficiency (Fig. 2B; Fig. S3B). Additionally, the incorporation of 1 or 2 mM H-Lys (Boc)-OH (BocK) and H-Lys (Aloc)-OH (AlocK) using *Mb*PylRS-349F was tested (38) (Fig. 2C; Fig. S3C) to reveal that the supplement of 2 mM AlocK exhibited the highest incorporation efficiency. Therefore, we chose the trc promoter to express *Mb*PylRS-349F and 2 mM AlocK for further experiments.

Considering the necessity of retaining native function after ncAA incorporation, we screened for permissive sites in Cro protein, which is composed of 66 amino acid residues. The first step was to identify sites throughout the whole Cro sequence where the AlocK can be inserted. A total of 14 sites were selected for this initial test (Fig. S5). Among those sites, K8, K18, K21, K32, K39, and K56 were selected because the natural amino acid K is structurally similar to AlocK (Fig. S2). Sites G24, V25, G37, R38, A46, and D47 were selected because they are located in the loop regions and do not interact with operator DNA (39). Sites F14 and E53 were selected because mutations at these sites were reported to have minimal impact on Cro activity (40). The native codons for these sites were individually mutated to TAG, yielding a series of recombinant plasmids pET26b-T5-CroNX (Fig. S1). Each pET26b-T5-CroNX was co-transformed with the plasmid expressing the *Mb*PylRS-349F/tRNA^Pyl^ (abbreviated as RSFT) pair into *E. coli* DH10B, followed by expression analysis of Cro variant with SDS-PAGE and simple western immunoblots. The results showed that only in the presence of AlocK, a protein band corresponding to the size of Cro was detected for variants Cro-8X, Cro-14X, Cro-21X, Cro-38X, Cro-39X, Cro-46X, Cro-53X, and Cro-56X, indicating the expression of a full-length Cro variant with AlocK incorporated at K8, F14, K21, R38, K39, A46, E53, and K56 in response to the TAG codon (Fig. 2D; Fig. S6A, S7A, and S7B). The ncAA incorporation efficiency at each site was assessed based on relative intensities detected by simple western immunoblots (Fig. 2D; Fig. S7B), revealing that K8, F14, K21, K39, A46, and E53 sites exhibited relatively higher incorporation efficiency (Fig. S7C). According to the resolved structure of Cro (39), the α2 (residues 16 to 23) and α3 (residues 27 to 36) helices directly interact with DNA, suggesting that at least the 36 amino acid residues at N-terminus are required for function. There is no study reporting whether the C-terminus of Cro is critical for its activity. To avoid the situation that the truncated Cro produced by the TAG mutation near the C-terminus might retain activity and interfere with functional assessment of the Cro-ncAA variants, we selected K8, F14, K21, and K39 from those sites with confirmed AlocK incorporation for subsequent functional assays.

Because the wild-type Cro represses the P_RM_ promoter and the expression of the downstream genes, and the P_RM_ promoter is weak without CI protein activation (41), we designed two constructs to evaluate the repression of the P_RM_ promoter by any Cro-ncAA variant (Fig. S8). Firstly, the reporter gene *mNG* and sequence encoding wild-type Cro or the variant Cro-8X were assembled downstream of promoter P_RM_ and P_R_, respectively, and inserted into a high-copy number plasmid vector with a ColE1 origin of replication (ori). The resulting plasmid, pET26b-RRM-Cro/Cro-8X-mNG, was transformed into *E. coli* DH10B, followed by mNG fluorescence measurement with flow cytometry (Fig. S1 and S8A). Because the Cro protein functions to inhibit the P_RM_ promoter and the mNG expression, it is reasoned that the truncated non-functional Cro-8X variant would lead to a strong fluorescence signal. However, the observed fluorescence intensity for the Cro-8X variant was very close to that for the wild-type Cro, suggesting that this design cannot provide sufficient resolution for the function assay (Fig. S8A). Second, we assembled a signal-amplifying module composed of sequences encoding the T7 RNA polymerase (T7RNAP) under the P_RM_ promoter and the *mNG* under the T7 promoter into the genome of the *E. coli* DH10B (Fig. S8B). The resulting strain, DH10B-T7RNAP-mNG, did not exhibit an increased fluorescence intensity when compared with the control strain *E. coli* DH10B, indicating the strength of the P_RM_ promoter cannot be detected using this system. Alternatively, the signal-amplifying module was cloned into a vector to yield the plasmid pUltra-trc-RSFT-T7mNG, which was co-transformed with pET26b-T5-CroNX into *E. coli* DH10B for function test (Fig. 2E; Fig. S1). The fluorescence intensities of the strain expressing the wild-type Cro or the truncated Cro-8X variant were distinguishable with this design. Importantly, the flow cytometry analysis showed that all the Cro variants with AlocK inserted at K8, F14, K21, and K39 sites exhibited repression of the P_RM_ promoter, with the Cro-K8AlocK variant exhibiting the strongest repression effect despite not being as strong as the wild-type Cro (Fig. 2E; Fig. S3D and S9). As the K8 residue of the Cro protein does not directly interact with the operator DNA (39), incorporation of AlocK at this position would minimally affect its function. In addition to AlocK, BocK could also be incorporated into Cro to repress the mNG expression at levels comparable to or lower than that of the Cro-K8AlocK variant (Fig. S10). Therefore, the Cro-K8AlocK variant was selected for further experiments.

### AlocK-induced prophage-mediated protein release

We aimed to construct a bacteria-based delivery system where the ncAAs lead λ phage into the lytic cycle, thereby controlling host bacteria lysis and releasing POIs (e.g., therapeutic proteins) expressed within the host. In this system, ncAAs act as effective inducers for controlled protein release, and a key step is to enable prophage activation with ncAAs.

After confirming the function of Cro-ncAA variants in heterologous host *E. coli* DH10B, we tested the feasibility of using ncAAs to induce prophage in the λ lysogen *E. coli* K12 WK 6λ. We first checked whether the λ lysogen contains any endogenous UAG suppressor by transforming it with plasmids expressing the reporter mNG-42X and an orthogonal aaRS/tRNA pair (Fig. S1). No fluorescence signal was observed in cells without the supplementation of the ncAA, in contrast to the intense fluorescence from cells with ncAA supplementation (Fig. S11), indicating the absence of a natural UAG suppression. Expressing the orthogonal RSFT pair or adding AlocK in the growth medium did not lead to prophage induction in the λ lysogen (Fig. S12). Moreover, expression of the wild-type Cro under the T5 promoter in the λ lysogen resulted in an increased number of prophages entering the lytic cycle (Fig. S13), confirming prophage induction by wild-type Cro overexpression (30). Collectively, these results support the feasibility of achieving prophage induction by Cro-ncAA, instead of UV or mitomycin C, which is not feasible for *in vivo* use.

**Fig 3:**
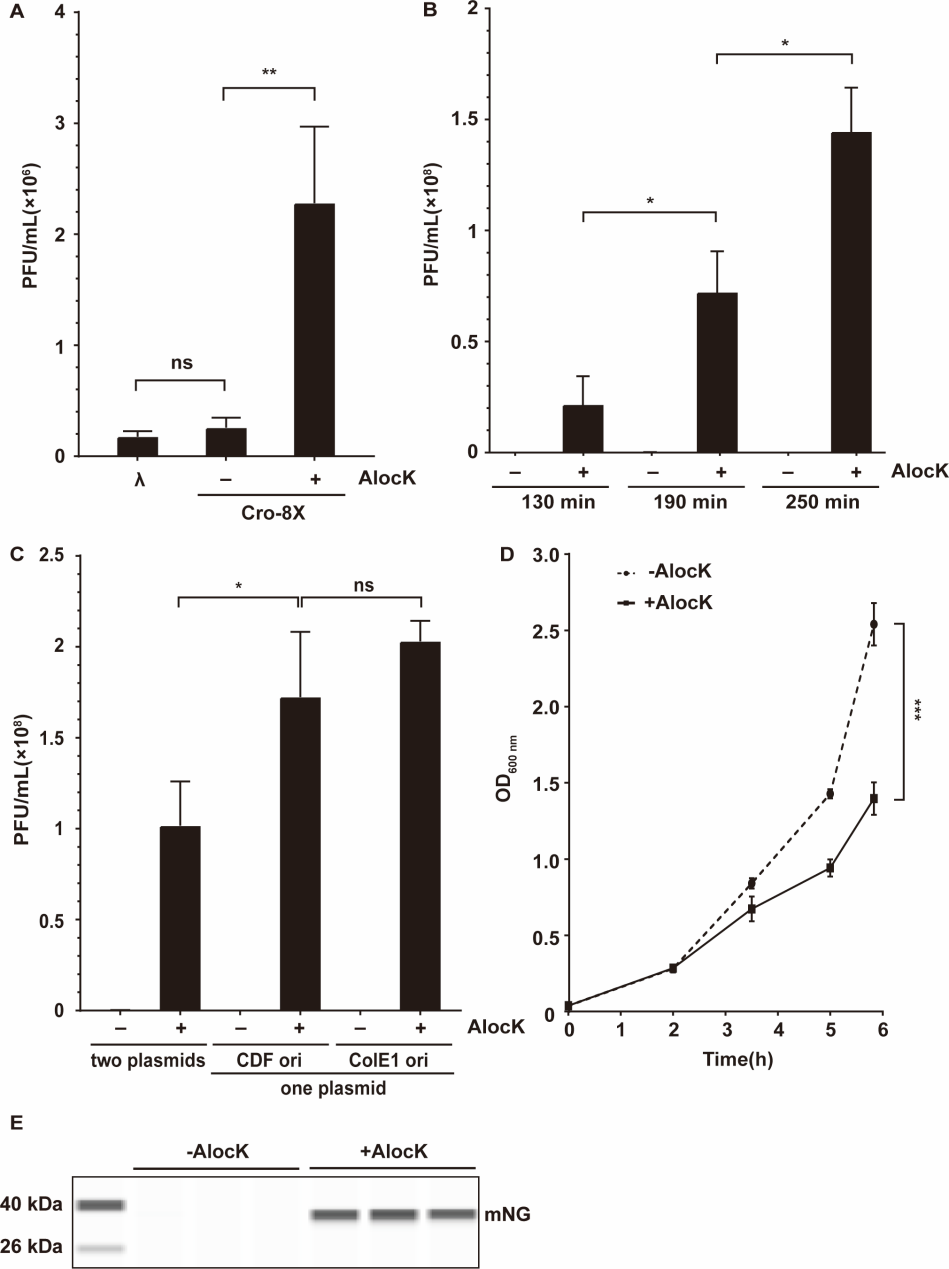
AlocK-mediated prophage induction and protein release. (A) Prophage induction by the Cro-K8AlocK variant. The incorporation of AlocK at the K8 site led to the expression of the functional Cro-K8AlocK and the activation of prophages. (B) Prophage induction over an extended induction time with AlocK. (C) Effect of expressing Cro-8X and RSFT separately in two plasmids or together in one plasmid on prophage induction. For panels A–C, the λ phage titers (PFUs/mL) were quantified using *E. coli* C600 CR34 as the recipient strain. (D) Growth curve of the λ lysogen that expresses Cro-8X and RSFT in one plasmid in the presence or absence of AlocK. For panels A–D, three independent biological replicates were performed with the mean and standard deviation presented. Two-tailed *t*-tests were performed to compare mean differences. *P* values indicated are as follows: ***P < 0.001; **P < 0.005; *P < 0.05; ns. not significant. (E) Detection of mNG released *via* prophage induction-mediated host lysis in the presence of AlocK. Three independent biological replicates were analyzed with simple western immunoblotting. The molecular weight of mNG is 26.7 kDa.

Next, we tested prophage induction in λ lysogen by the Cro-ncAA variant that requires ncAAs for full-length Cro expression and proper function. When the λ lysogen strain carrying pET26b-T5-Cro8X (with ColE1 ori) and pUltra-trc-RSFT (with CDF ori) (Fig. S1) was provided with IPTG and AlocK, an increasing amount of λ phages was detected in the medium, indicating more prophages entered the lytic cycle (Fig. 3A). As IPTG alone did not lead to more phages, this result indicated that the Cro-K8AlocK variant induced the prophage. To eliminate the requirement for IPTG, we replaced the inducible T5 promoter for Cro-8X with the constitutive promoter 1.8 (42) and the inducible trc promoter for *Mb*PylRS-349F with a constitutive trc promoter by removing the lac operator and re-tested the effect of AlocK on prophage induction (Fig. 3B; Fig. S1). An increasing number of phages were detected when the AlocK induction time was extended from 130 to 250 min, suggesting that the accumulation of the Cro-K8AlocK variant induced more prophages entering the lytic cycle. To simplify the system, we assembled genes encoding the Cro-8X and RSFT into a single recombinant plasmid (pUltra-CRSFT-mNG with a CDF ori and pUltra-ColE1-CRSFT-mNG with a ColE1 ori), which led to the release of more phages (Fig. 3C; Fig, S1). Moreover, the λ lysogen carrying pUltra-CRSFT-mNG grew much slower when supplemented with AlocK, suggesting the occurrence of prophage induction-mediated cell lysis (Fig. 3D).

To detect POI release upon host cell lysis, the λ lysogen carrying the plasmid pUltra-CRSFT-mNG for expression of Cro-8X, RSFT, and reporter protein mNG (strain λCFTM) was fed with or without AlocK, followed by analysis of the proteins released into the growth medium *via* SDS-PAGE and simple western immunoblotting. The experimental results confirmed the release of intracellular proteins and, notably, mNG in the medium supplemented with AlocK (Fig. 3E; Fig. S6B and S14). Additionally, BocK could be incorporated at the K8 site of Cro to induce prophage activation and protein release (Fig. S15), demonstrating the versatility of ncAA selection in this system. Overall, these results indicate that the ncAA triggers prophage induction and release of proteins expressed in the host (i.e., protein release *via* prophage induction-mediated bacteria lysis) and acts as an inducer to control protein release in the bacteria-based delivery system.

### Evaluation of the bacteria-based delivery system in mice

**Fig 4:**
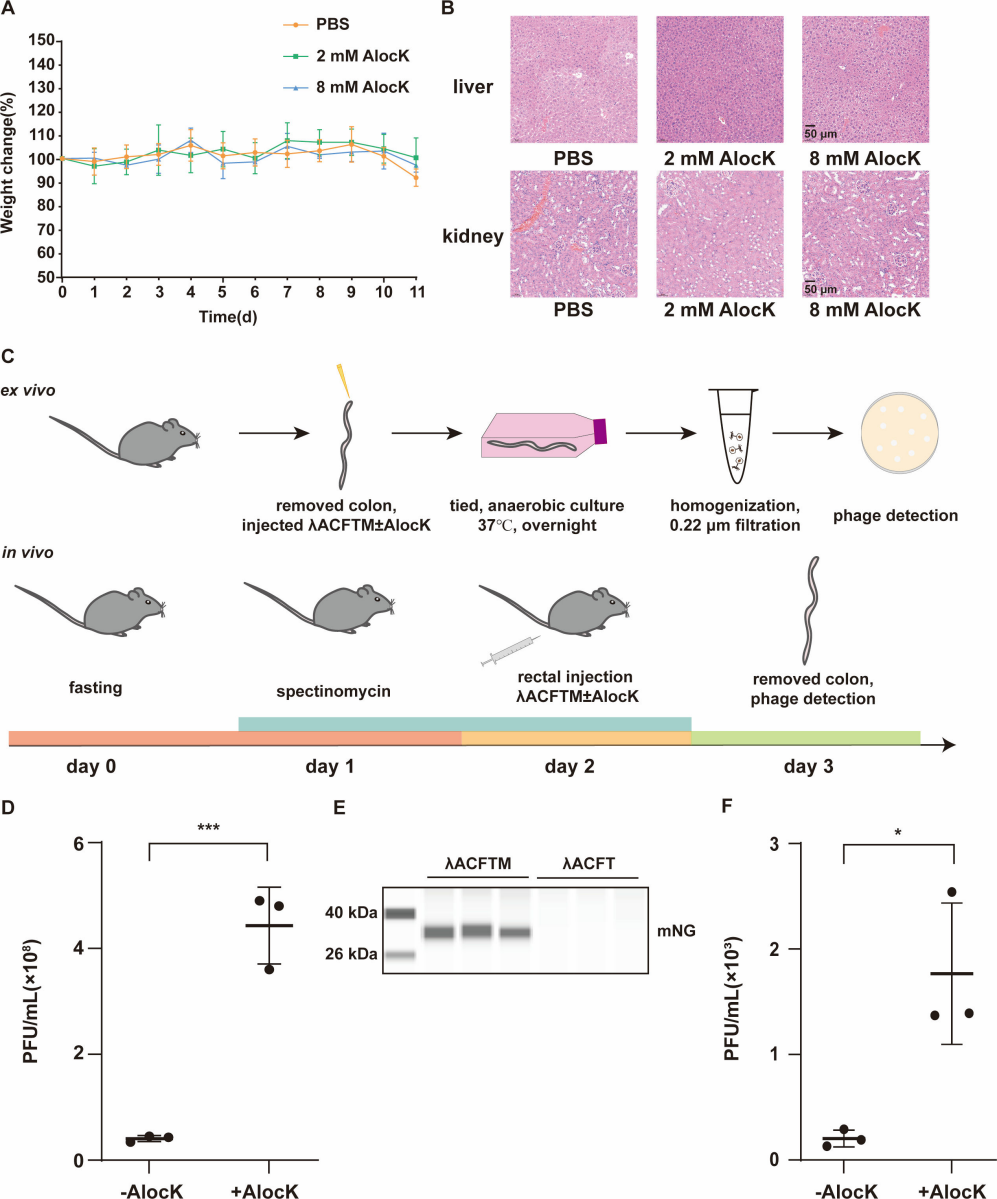
Assessment of protein release due to prophage induction with AlocK *ex vivo* and *in vivo*. (A and B) Safety assay of AlocK in mice. (A) Body weights of mice receiving daily oral gavage of 1× PBS (orange), 2 mM AlocK (green), or 8 mM AlocK (blue) for 11 days. The initial weight was set as 100%. *n* = 5. (B) Hematoxylin and eosin staining images of liver and kidney from mice treated with PBS or AlocK. (C) A scheme illustrating the detection of AlocK-induced prophages *ex vivo* and *in vivo*. For detection *ex vivo*, the colons of healthy mice were collected and injected with λACFTM cells only or with an AlocK supplement. After a 16 h cultivation, the colons were processed for phage titer detection. For detection *in vivo*, fasted mice received rectal administration of λACFTM cells only or with an AlocK supplement. The colons were collected for phage titer detection. (D) and (F) show the titers of phages detected *ex vivo* and *in vivo*, respectively. Mean and standard deviation of the number of plaques (PFUs/mL) were presented. Two-tailed *t*-tests were performed to compare mean differences; *P* values indicated are as follows: ***P < 0.001; *P < 0.05. (E) Release of mNG in the murine gut environment *via* prophage induction-mediated host lysis in the presence of AlocK. Three independent biological replicates were performed and analyzed with simple western immunoblotting.

We explored the application of this bacteria-based delivery system in animal models for protein release purposes. First, the toxicity of AlocK in mice was evaluated. The C57BL/6J mice were orally administered with phosphate-buffered saline (PBS), 2 mM AlocK, or 8 mM AlocK (prepared in PBS) daily for 11 days. There was no significant difference in body weight changes (an indicator of health to assess the *in vivo* toxicity) (6) between the three groups (Fig. 4A). On day 12, blood samples and organs, including liver and kidney, were collected for further examination. No significant difference in routine blood test results was observed for the mice treated with PBS or 8 mM AlocK (Fig. S16). The hematoxylin–eosin (HE) staining results indicated no obvious differences in the liver and kidney tissues among the three groups (Fig. 4B). All these results demonstrate that AlocK is safe for use *in vivo*.

Next, we proceeded to test whether the bacteria-based delivery system could function in the complex colon environment (43, 44) (Fig. 4C). The colons of healthy C57BL/6J mice were collected and injected with 1 × 10^9^ CFUs of *E. coli* K12 WK 6λ *ea47::ampR* (strain λA) carrying plasmid pUltra-CRSFT-mNG (strain λACFTM), with or without AlocK, followed by anaerobic incubation in Dulbecco’s modified Eagle’s medium supplemented with 10% fetal bovine serum at 37°C. Phage titers were determined after processing the colons with the contents. A higher phage titer was observed with the AlocK supplement (Fig. 4D), indicating that AlocK induces prophages in the murine gut environment. To further detect the release of proteins due to prophage induction-mediated bacterial cell lysis, we adopted a similar approach by injecting the colons of healthy C57BL/6J mice with AlocK solution and 1 × 10^9^ CFUs of λACFTM or λACFT (a strain differing from λACFTM in lacking the *mNG* gene) as a control. The protein fraction was precipitated with acetone after processing the colons with the contents, followed by simple western immunoblotting to detect the released mNG. The mNG protein was only detectable in the samples receiving λACFTM and AlocK (Fig. 4E; Fig. S6C), demonstrating that AlocK can induce POI release in the murine gut environment. Subsequently, we tested whether the system could function *in vivo*. Two groups of mice received a rectal administration of λACFTM (1 × 10^9^ CFUs) only or together with AlocK, and after euthanization on the next day, their colons were collected and processed as previously described to quantify phage titers (Fig. 4C). The group receiving AlocK showed a higher phage titer (Fig. 4F), indicating that AlocK induces prophages *in vivo*. These findings demonstrate that the AlocK can function as an inducer for regulating protein release in the bacteria-based delivery system in the murine gut environment and promote λ prophage induction *in vivo*, indicating its potential as an inducer for bacteria-based delivery systems *in vivo*.

### Constitutive protein expression by AlocK induction in bacteria-based delivery systems

**Fig 5:**
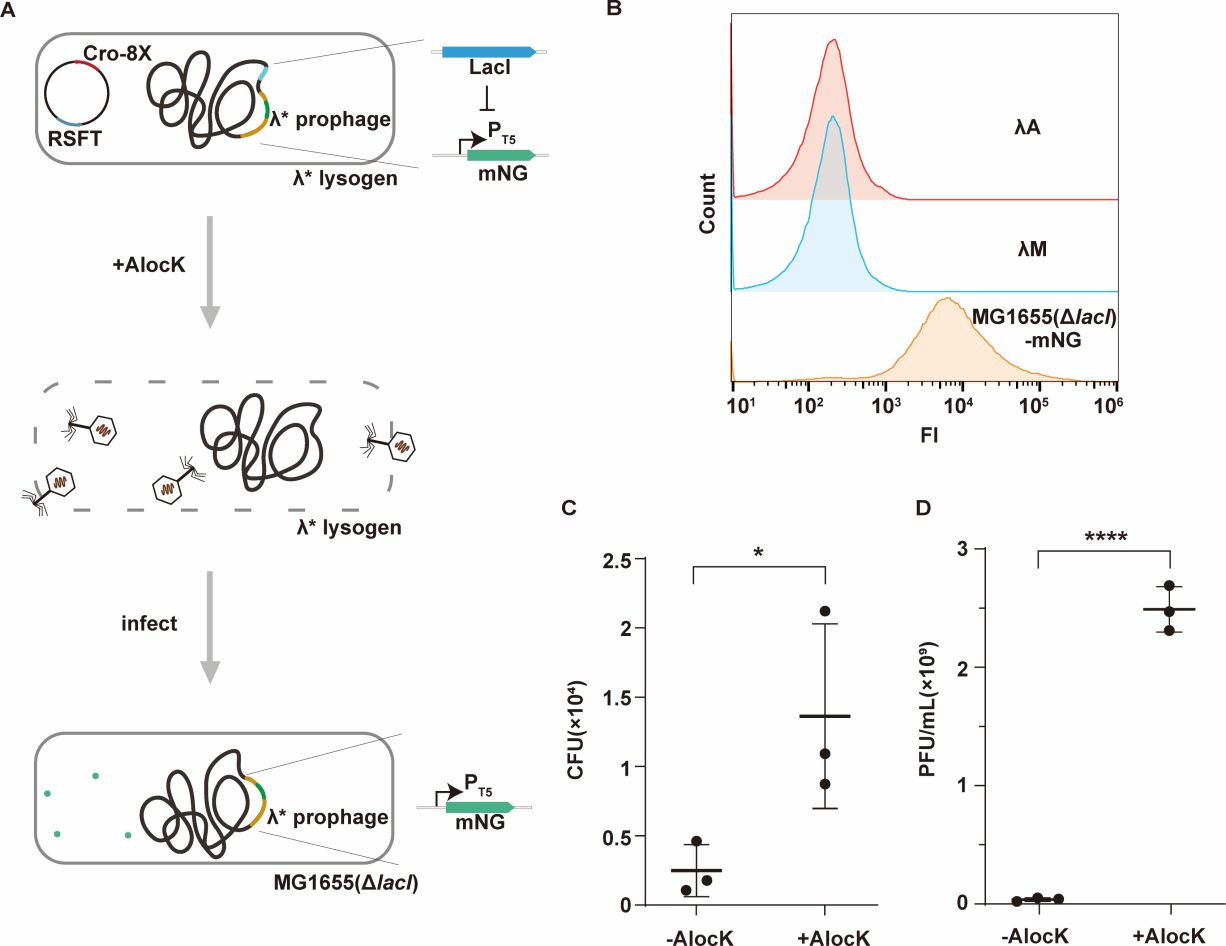
Detection of continuous protein expression induced by AlocK. (A) A scheme illustrating protein expression by AlocK induction. The reporter gene *mNG* driven by a T5 promoter was inserted into the λ prophage genome. In the resulting λ* lysogen, high levels of LacI repress mNG expression. The addition of AlocK leads to the expression of the Cro-K8AlocK variant and the transition of λ* prophage into the lytic cycle. As the released phage progenies integrate into the recipient strain *E. coli* MG1655 *lacI::cmR*(Δ*lacI*), repression of the T5 promoter is released, leading to expression of mNG (green dots). (B) Detection of mNG expression in the recipient *E. coli* strain with flow cytometry. MG1655(Δ*lacI*)-mNG: the recipient strain with λ* phage integrated into the genome. λA: strain *E. coli* K12 WK 6λ *ea47::ampR*. λM: strain *E. coli* K12 WK 6λ *ea47::mNG*. Both λA and λM serve as negative controls. (C) Detection of the integration of λ* phage into the *E. coli* MG1655 *lacI::cmR* cells in the *ex vivo* assay. The numbers of CFUs were presented as the mean with standard deviation (SD). (D) Detection of λ* phages released upon AlocK induction in the *ex vivo* assay. The numbers of plaques (PFUs/mL) were presented as the mean with SD. Two-tailed *t*-tests were performed to compare mean differences; *P* values indicated are as follows: ****P < 0.0001; *P < 0.05. FI: fluorescence intensity. mNG: mNeonGreen.

Next, we extended the application of AlocK to achieve constitutive POI expression in bacteria-based delivery systems. The reporter gene *mNG* under the T5 promoter was integrated into the non-essential gene *ea47* in the λ prophage genome (28), resulting in the strain *E. coli* K12 WK 6λ *ea47::mNG* (strain λM). In *E. coli* K12 WK 6λ, the T5 promoter is repressed due to its native LacI protein expressed at high levels (Fig. 5A). Upon prophage induction by AlocK, the λ phage progenies deliver the *mNG* gene into a provided recipient strain (e.g., *E. coli* MG1655 *lacI::cmR*) (45), leading to mNG expression due to the lack of LacI in the recipient strain. In this case, AlocK acts as an inducer for continuous protein expression (Fig. 5A). To test this, we expressed Cro-8X and RSFT in the strain λM and incubated the phage progenies released with *E. coli* MG1655 *lacI::cmR*. The resulting cells exhibited a distinct fluorescence signal of mNG compared with control strains λM and λA (Fig. 5B; Fig. S3E). Whether AlocK could induce λ prophage induction and λ phage genome integration into the provided recipient *E. coli* strain in the murine gut environment was also tested. A mixture of 5 × 10^8^ CFUs of λM expressing Cro-8X and RSFT and 5 × 10^8^ CFUs of *E. coli* MG1655 *lacI::cmR* supplemented with or without AlocK was injected into colons collected from healthy mice. After anaerobic cultivation, the colon samples were homogenized and filtered through 5 µm filters. Considering that the λ phage genome and the *E. coli* MG1655 *lacI::cmR* strain contain the gene conferring resistance to ampicillin and chloramphenicol, respectively, we screened for colonies of *E. coli* MG1655 *lacI::cmR* integrated with the λ phage genome on plates containing both ampicillin and chloramphenicol (Fig. 5C). The phage titer was measured using the aforementioned methods concurrently. The results indicated that the addition of AlocK led to the induction of more phages (Fig. 5D), which could integrate into the *E. coli* MG1655 *lacI::cmR* genome. Collectively, these results demonstrate that AlocK can induce prophage and, due to the reinfection of λ phage into the provided recipient *E. coli* strain, can be utilized for sustained protein expression in bacteria-based delivery systems.

### Independent regulation of different proteins with ncAAs

**Fig 6:**
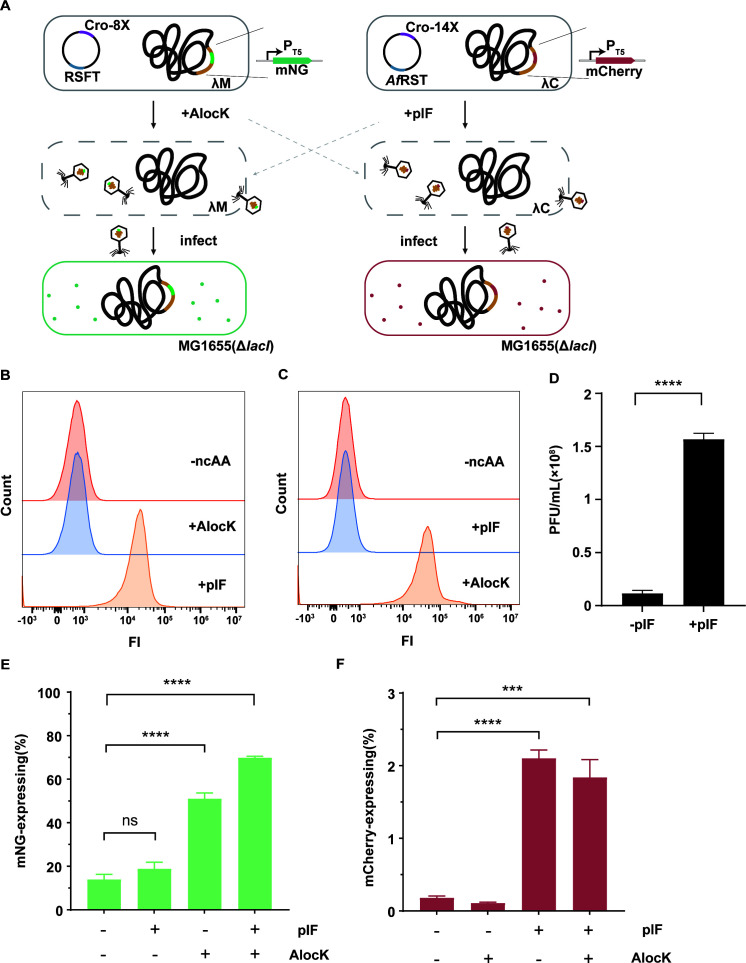
Different ncAAs induce prophages and activate protein expression independently and simultaneously. (A) Scheme of independent regulation of different proteins of interest with mutually orthogonal aaRS/tRNA pairs and their cognate ncAAs. The AlocK and pIF are specifically recognized by *Mb*PylRS-349F/tRNA^Pyl^ (RSFT) and *Af*pIFRS/tRNA^Tyr^ (*Af*RST) to induce lysis of the engineered λ lysogens λM and λC, respectively. Released phages deliver the reporter gene into the recipient strain *E. coli* MG1655 (Δ*lacI*), leading to an expression of mNG or mCherry. The gray dash arrow indicates no crosstalk between AlocK and pIF. (B and C) Orthogonality assay of RSFT and *Af*RST. Using sfGFP-39X as the reporter, incorporation of AlocK and pIF by *Af*RST (B) and RSFT (C) was tested. (D) Prophage induction by the pIF-containing variant Cro-F14pIF. The phage titers (PFUs/mL) in the absence and presence of pIF were quantified. (E and F) Independent protein expression activation with AlocK and pIF. Phages released under four different treatments (no ncAA, AlocK, pIF, a mixture of AlocK, and pIF) were incubated with *E. coli* MG1655 (Δ*lacI*), followed by flow cytometry to count cells expressing mNG (E) or mCherry (F). “mNG-expressing” and “mCherry-expressing” refer to the percentage of bacterial cells that express mNG or mCherry, respectively. Two-tailed *t*-tests were performed to compare mean differences; *P* values indicated are as follows: ****P < 0.0001; ***P < 0.001; ns, not significant.

After confirming the regulation of the reporter mNG by AlocK, we next explored independent regulation of different POIs by mutually orthogonal aaRS/tRNA pairs and their cognate ncAAs (Fig. 6A). We started by testing the orthogonality between the *Af*pIFRS/tRNA^Tyr^ (*Af*RST) and RSFT pairs. Fluorescence of the reporter protein sfGFP-39X in the *E. coli* DH10B cells expressing *Af*RST (Fig. S1) in the presence or absence of 1 mM ncAA (pIF or AlocK) was measured by flow cytometry. Intense fluorescence was detected only in the pIF-supplemented sample (Fig. 6B; Fig. S3F), indicating *Af*RST recognizes pIF but not AlocK. Meanwhile, the cells expressing RSFT exhibited intense fluorescence only in the presence of AlocK (Fig. 6C; Fig. S3G), indicating RSFT recognizes AlocK but not pIF. These results demonstrate the mutual orthogonality between the pIF-specific *Af*RST and AlocK-specific RSFT. Moreover, the function assay results showed that pIF can be incorporated to produce functional Cro-pIF variants (Fig. S17), and the Cro-14X variant exhibiting the best performance was used to test prophage induction with pIF. When the engineered λ lysogen expressing both *Af*RST and the Cro-14X variant was supplied with pIF, a significant number of released phages was detected (Fig. 6D).

The results above collectively imply that pIF and AlocK can be applied to induce bacterial lysis and regulate two POIs independently. To prove this, we individually transformed engineered λ lysogen strains, λM and λC, which carry the reporter gene *mNG* or *mCherry* in the λ prophage genome, with the plasmid expressing RSFT and Cro-8X or *Af*RST and Cro-14X (Fig. 6A). The resulting λ lysogen strains were mixed in equal cell number and induced under four different conditions: 0 mM ncAA, 2 mM AlocK, 2 mM pIF, and a mixture of 2 mM AlocK and 2 mM pIF. The phages released under these four treatments were collected and incubated with *E. coli* MG1655 *lacI::cmR*, followed by flow cytometry to count cells exhibiting fluorescence of mNG or mCherry. The detection of mNG or mCherry fluorescence indicates successful delivery and expression of the reporter gene in the recipient *E. coli* cell. As shown in Fig. 6E, cells expressing mNG significantly increased upon induction with AlocK, while no effect was observed with pIF induction. This demonstrates that AlocK successfully and specifically induced the prophage in the λM strain and further triggered mNG expression. Meanwhile, pIF, not AlocK, led to a significantly increasing number of mCherry-expressing cells (Fig. 6F), indicating successful and specific prophage induction in the λC strain and subsequent mCherry expression. A supplement of both AlocK and pIF resulted in increasing numbers of cells expressing mNG or mCherry, demonstrating that the addition of the two ncAAs allows for concurrent expression of two proteins. Overall, these results illustrate that the use of two ncAAs enables the independent regulation of two POIs, which provides a new method for the independent control of multiple proteins in combination therapy.

## DISCUSSION

In this work, we applied ncAAs to regulate the lifecycle of λ phage, thereby establishing a ncAA-induced protein regulation system for bacteria-based delivery. By testing aaRS/tRNA pairs, promoters of aaRS, and ncAAs, we found that expression of *Mb*PylRS-349F by the trc promoter led to the best ncAA incorporation efficiency with 2 mM AlocK. Screening for permissive sites of Cro revealed that AlocK was efficiently incorporated to give functional Cro variants. In the λ lysogen strain engineered to express the *Mb*PylRS-349F/tRNA^Pyl^ pair and Cro-8X, the supplement of AlocK successfully induced the prophage and, consequently, one-time protein release due to host cell lysis. In this case, prophage induction relies solely on AlocK, bypassing the need for host SOS responses. When the gene encoding POI is inserted into the λ prophage genome, AlocK can further trigger continuous POI expression in an engineered *E. coli* MG1655 recipient strain. We also confirmed that this bacteria-based delivery system retains the ability of AlocK to induce λ prophage in mice and trigger protein release in the gut environment. In addition, we demonstrated that another ncAA, pIF, can also be incorporated by *Af*pIFRS into Cro protein for protein regulation purposes. Importantly, AlocK and pIF act to regulate different mNG and mCherry independently and simultaneously. This study pioneers the use of ncAAs to regulate phage life cycles and control protein release and expression in bacteria-based delivery systems.

Built on genetic code expansion, this strategy is simple and convenient, requiring minimal genetic engineering efforts other than introducing an orthogonal aaRS/tRNA pair and an amber TAG mutation in Cro protein. In the proof-of-concept study, fluorescent proteins (mNG and mCherry) were adopted as reporters to illustrate the regulation of POI expression and release by ncAA-controlled prophage induction. Many components in this system can be easily substituted to suit various applications. First, depending on whether the *POI* gene is integrated into a plasmid or the prophage genome, this strategy provides options for both single-time and long-term protein drug delivery, catering to a range of therapeutic needs. Second, the reporter proteins can be readily replaced by therapeutic proteins. Since the amber codon is introduced in the Cro protein, the POI can be directly substituted without retesting the ncAA incorporation efficiency. The promoters of POI can also be adjusted to regulate expression levels and drug dosages. Targeted localization of POI may be achieved through fused expression with a signal peptide or membrane-anchored protein. Third, ncAAs can be substituted if needed. In the study, AlocK, BocK, and pIF have been shown to produce functional Cro-ncAA variants, demonstrating flexibility in ncAA selection. Furthermore, introducing mutually orthogonal aaRS/tRNA pairs enables independent regulation of multiple POIs with different ncAAs. Notably, an ncAA specifically induces targeted prophages in lysogens engineered to encode the corresponding orthogonal aaRS/tRNA pair, unlike general prophage inducers, such as mitomycin C. This offers a novel method for precisely controlling protein drugs with ncAA cocktails in combination therapy. Additionally, ncAAs are chemical synthetic analogs of natural amino acids, which should avoid cross-interaction with endogenous or environmental natural amino acids. Many ncAAs (e.g., AlocK, BocK, and OmeY) have been demonstrated to be safe for mice by ourselves and others (20, 46), and ncAAs are important building blocks of medical drugs (e.g., antibiotics, antibody-drug conjugates) (47, 48), which are in clinical use or trials. The application of ncAAs in biomedicine and biotherapeutics suggests that ncAAs can be safely used in animals and even humans.

Both the λ phage and *E. coli* MG1655 used in the bacteria-based delivery system are non-pathogenic model organisms and have been widely used in biomedicine and biotechnology, including as the chassis of genetically engineered microbes for disease treatment (45, 49–51). Due to its bacterial host specificity, the λ phage has been used as an alternative to antibiotics to kill enterohemorrhagic *E. coli* strains (50) and repress targeted genes in *E. coli* MG1655 (45) with minimal disruption to the gut microbiome (45, 50). Additionally, λ phage serves as a chassis of phage display technology for developing drugs, vaccines, and diagnostic tools (52). Unlike traditional phage-based delivery systems, the λ phage in our study can perform dual roles under the regulation of ncAAs: lysing bacteria and releasing POIs produced in the bacteria or delivering the *POI* gene specifically into a recipient strain for sustained POI expression, which expands the application of λ phage and confers multifunctionality to the bacteria-based drug delivery system. This system can be further optimized in several aspects. The Cro-ncAA variants showed reduced efficacy compared to wild-type Cro likely due to low ncAA incorporation in the λ lysogen. Studies have reported that knockout of the translation termination release factor RF1 or implementation of an orthogonal ribosome improves ncAA incorporation (53, 54). Similar efforts may be adopted to improve performance in our study. Genes encoding the aaRS/tRNA pairs can be integrated into the genome of λ lysogen for genetic stability. To assay the bacteria-based delivery system’s efficacy in mice, we first explored oral gavage for bacteria administration instead of rectal injection and obtained minimal detection of AlocK-induced phages. It is likely that the acidic gastric environment severely degrades the bacteria-based delivery system, and protective materials to ensure effective release in the intestinal environment would be beneficial.

Using a similar strategy, prophages and their bacteria hosts other than the *E. coli* phage λ model system may also be engineered as bacteria-based delivery systems, enabling disease-specific therapies based on the unique characteristics of each strain. One of these potential examples is that *Salmonella* spp., in which the genetic code expansion has been established (55), could be engineered to release therapeutic proteins directly in tumors using this strategy, leveraging their natural tumor tropism (2, 3). Notably, the lysis-based protein drug delivery with the clinically relevant *Salmonella* sp. in addition to non-pathogenic *E. coli* strains indicated that the residues of these engineered bacteria could be well-tolerant and cleared without obvious side effects in mice models (3, 56). When applying in non-model systems, it will be necessary to remove toxic genes and use phages with a narrow host spectrum. Furthermore, for biocontainment, a ‘life switch’ can be installed by introducing amber codons in genes essential for the phages and the bacterial hosts. Similar to inserting AlocK or pIF into Cro to regulate λ prophage life cycle, ncAAs can be inserted into tail fibers, DNA polymerase, or integrase to restrain phage proliferation only in the bacteria that can decode the amber codon. This strategy has already been applied to engineer bacteria and viruses with stringent growth dependence on ncAAs and strong resistance to evolutionary escape mechanisms, including horizontal gene transfer (21, 22, 24).

Additionally, our work provides a pioneering case study for applying genetic code expansion technology in bacteriophage biology research. Although prophage genomes have been identified in numerous bacteria through sequence analysis (57–59), the prophage induction and phage-bacteria interaction remain largely unresolved. The tools used in this work to insert ncAAs into Cro can be transferred to site specifically incorporating ncAAs with designed functions (e.g., photoreactive, fluorescent) into phage proteins to capture and monitor phage–bacteria interaction (60). Importantly, substitution with ncAAs is a small modification so that the native protein function can be well maintained.

In summary, we present a strategy using ncAAs as prophage inducers for protein regulation in bacteria-based delivery systems. This approach is simple and convenient to operate and adaptable to different scenarios, showing promise for *in situ* protein drug delivery and phage therapy, as well as phage biology research.

## MATERIALS AND METHODS

### Plasmids and strains

Cartoons of plasmids and strains used in this study are shown in Fig. S1; Table S1, respectively. Sequences of related proteins and promoters are listed in Table S2. The detailed process of plasmid and strain construction is summarized in Table S3, with the primers listed in Table S4.

All plasmids were constructed on pET-26b(+) (Novagen) and pUltra-*Mb*PylRS (61). The plasmid pEVOL-pAzF was obtained from Addgene (Addgene #31186). Gene fragments encoding mNeonGreen (mNG), super-folder GFP (sfGFP), *Af*pIFRS, *CMa*PylRS, *Af*tRNA^Tyr^, and *CMa*tRNA^Pyl^ were synthesized by Sangon Biotech. The DNA sequences encoding Cro and T7 RNA polymerase were amplified from the genome of *E. coli* strains K12 WK 6λ and BL21(DE3), respectively. DNA fragments were assembled using the ClonExpress MultiS One Step Cloning Kit (Vazyme). Site-directed mutagenesis of the gene encoding Cro, mNG, sfGFP, or *Mb*PylRS was performed using Mut Express II Fast Mutagenesis Kit V2 (Vazyme). The *E. coli* DH5α was used as the host for molecular cloning. The concentration of antibiotics used was 15 µg/mL for chloramphenicol, 25 µg/mL ampicillin, and 50 µg/mL for kanamycin and spectinomycin, unless specified otherwise. All recombinant plasmids were confirmed by DNA sequencing.

Strains *E. coli* DH10B and DH10B-T7RNAP-mNG were used to test ncAA incorporation efficiency and the function of Cro-ncAA. The strain DH10B-T7RNAP-mNG was constructed *via* λ-Red mediated recombineering (62) to insert the gene encoding T7 RNA polymerase (under P_RM_ promoter), *mNG* (under the T7 promoter), and an ampicillin resistance gene (*ampR*) upstream of the *lacY* gene in the genome of *E. coli* DH10B. Three *E. coli* K12 WK 6λ mutants, *ea47::ampR*, *ea47::mNG,* and *ea47::mCherry*, were constructed for expression tests of genes integrated into the λ phage genome by replacing the *ea47* gene with the *ampR* or a cassette containing both the *ampR* and either the *mNG* or the *mCherry* (under T5 promoter). The strain *E. coli* MG1655 *lacI::cmR* was used as the recipient for phage integration to trigger continuous protein expression and constructed by replacing the *lacI* gene with a chloramphenicol resistance gene (*cmR*).

### Comparison of incorporation efficiencies of orthogonal translation systems

Three colonies were picked randomly and individually inoculated in Luria–Bertani (LB) medium with antibiotics, 1 mM IPTG, 0.02% (w/v) arabinose, and different concentrations (0, 1, and 2 mM) of a ncAA. The ncAAs used in this study include H-Lys(Boc)-OH (BocK; CAS: 2418-95-3; Bidepharm), 4-Iodo-L-phenylalanine (pIF; CAS: 24250-85-9; Energy Chemical), 4-Azido-L-phenylalanine (pAzF; CAS: 33173-53-4; Meryer), and H-Lys(Aloc)-OH (AlocK; CAS: 6298-03-9; Macklin). Cells that have been cultivated at 37°C with shaking (200 rpm) for 24 h were collected, and their fluorescence was measured with flow cytometry.

The combinations of plasmids and ncAAs used to compare ncAA incorporation efficiency are summarized in Table 1. Combinations 1–4 were used to test the incorporation efficacy of different aaRS/tRNA pairs. Combinations 5 and 6 were used to test the incorporation efficacies of *Mb*PylRS-349F and *Af*pIFRS, and combinations 7 and 8 for *Mb*PylRS-349F and pAzFRS. Combinations 7, 9, and 10 were used to test the incorporation efficacies of different promoters for aaRS. Combinations 5, 11, 12, and 13 were used to test the incorporation efficacy with different ncAA supplements. Combinations 12, 14, 15, and 16 were used to test the orthogonalities of *Mb*PylRS-349F and *Af*pIFRS. tacI, glnS, lacUV5, and ara refer to the tacI, glnS, lacUV5, and araBAD promoters, respectively.

**TABLE 1:** Combinations of plasmids and ncAAs used for incorporation efficiency assay.

Combination	Plasmid 1	Plasmid 2	ncAA	Concentration of ncAA
1	pUltra-*Mb*PylRS	pET26b-T5-mNG42X	BocK	1 mM
2	pUltra-chPylRS-IPYE	pET26b-T5-mNG42X	BocK	1 mM
3	pUltra-tacI-RSFT	pET26b-T5-mNG42X	BocK	1 mM
4	pUltra-*CMa*RST	pET26b-T5-mNG42X	BocK	1 mM
5	pUltra-glnS-RSF	pET26b-ara-sfG39X-T	BocK	1 mM
6	pUltra-glnS-*Af*RST	pET26b-ara-sfG39X	pIF	2 mM
7	pUltra-trc-RSFT	pET26b-T5-mNG42X	BocK	1 mM
8	pEVOL-pAzF	pET26b-T5-mNG42X	pAzF	1 mM
9	pUltra-lacUV5-RSFT	pET26b-T5-mNG42X	BocK	1 mM
10	pUltra-tacI-RSFT	pET26b-T5-mNG42X	BocK	1 mM
11	pUltra-glnS-RSF	pET26b-ara-sfG39X-T	BocK	2 mM
12	pUltra-glnS-RSF	pET26b-ara-sfG39X-T	AlocK	1 mM
13	pUltra-glnS-RSF	pET26b-ara-sfG39X-T	AlocK	2 mM
14	pUltra-glnS-RSF	pET26b-ara-sfG39X-T	pIF	1 mM
15	pUltra-glnS-*Af*RST	pET26b-ara-sfG39X-T	pIF	1 mM
16	pUltra-glnS-*Af*RST	pET26b-ara-sfG39X-T	AlocK	1 mM

### Flow cytometry

The cell pellet from the collected culture was resuspended in phosphate-buffered saline (PBS) buffer, filtered through a 300-mesh nylon screen, and analyzed using a CytoFLEX (Beckman) flow cytometer. The sample flow rate was 30 µL per minute for detecting either sfGFP/mNeonGreen with a blue laser (wavelength = 488 nm) or mCherry with a yellow laser (wavelength = 561 nm) and 10 µL per minute for detecting both simultaneously. Data were collected for 120 s or until 100,000 events were recorded, whichever occurred first. Data analysis was performed using FlowJo software.

### Automated western immunoblotting

The simple western immunoblots were performed on Jess (ProteinSimple) using the Size Separation Master Kit with Split Buffer (2–40 kDa) according to the manufacturer’s standard instruction using the following antibodies: anti-his-tag antibody (GNI 4310-HS; Shanghai Genomics Technology, Ltd.) and mNeonGreen rabbit pAb (A24858; ABclonal). The Compass for Simple Western (version 6.0.0) software was used to analyze and present the simple western immunoblots.

### Expression and function assay of Cro-ncAA variants

To evaluate the strength of the P_RM_ promoter for function analysis of the Cro-ncAA variants, colonies of *E. coli* DH10B, DH10B-T7RNAP-mNG, or the strain carrying the plasmid pET26b-RRM-Cro-mNG or pET26b-RRM-Cro8X-mNG were cultured at 37°C for 24 h, and samples were collected for flow cytometry analysis.

To assess the function of the AlocK-containing Cro variants, the *E. coli* DH10B strain carrying both pUltra-trc-RSFT-T7mNG and pET26b-T5-CroNX was cultured at 37°C and 200 rpm for 12 h in LB medium with antibiotics, and then sub-cultured (1:100) into fresh LB medium with antibiotics. When the OD_600_ reached 0.6–0.8, the culture was supplemented with 1 mM IPTG, 0 mM, or 2 mM ncAA and further cultivated for 5 h. Cultures that were not supplemented with IPTG served as controls. Samples were collected for fluorescence measurement with flow cytometry as described above. Meanwhile, 1.8 × 10^7^ cells were collected, and the lysate was subjected to Tricine–SDS-PAGE (63) and simple western immunoblots analysis to detect the expression of Cro-ncAA. The expression levels of Cro-ncAA variants were calculated with the Compass for Simple Western. In addition, plasmids pUltra-trc-*Af*RST-T7mNG and pET26b-T5-CroNX were transformed into *E. coli* DH10B for the function test of the pIF-containing Cro variants following the assay methods described above.

### Prophage induction and protein release by ncAA

The plasmid pUltra-CRSFT-mNG for expression of *Mb*PylRS-349F/tRNA^Pyl^, mNG, and the Cro-8X mutant was transformed into *E. coli* K12 WK 6λ. Single colonies were inoculated into LB medium containing spectinomycin and grew at 37°C and 200 rpm for 12 h. The resulting culture was diluted (1:100) into LB medium with 2 mM AlocK and spectinomycin and cultivated at 37°C and 200 rpm for 4.5 h. A total of 4 × 10^7^ cells were collected, brought up to 1 mL with LB medium, and centrifuged at 4°C and 6,000 rpm for 3 min. The supernatant was filtered through a 0.22 µm membrane (Millipore), and the phage titers were determined using the double-layer agar method. Specifically, a 100 µL aliquot was mixed with the recipient *E. coli* C600 CR34 (1.2 × 10^7^ CFUs in 100 µL), followed by incubation at 37°C for 30 min. The mixture was diluted with 5 mL prewarmed top agar (LB medium with 0.5% agar) and poured over LB solid plates. The numbers of plaques appearing on plates after cultivation at 37°C for 12 h were counted. In addition, the proteins in the filtered samples were precipitated with cold acetone and analyzed with simple western immunoblots to detect mNG. The plasmids pUltra-trc-*Af*RST and pET26b-T5-Cro14X were transformed into the *E. coli* K12 WK 6λ *ea47::ampR* strain to test prophage induction by pIF following the same steps described above. ncAA induced continuous protein expression.

The plasmid expressing *Mb*PylRS-349F/tRNA^Pyl^ and the Cro-8X mutant was transformed into *E. coli* K12 WK 6λ *ea47::mNG*. Single colonies were inoculated into LB containing spectinomycin and cultured at 37°C and 200 rpm for 12 h. The culture was diluted (1:100) into LB medium supplemented with 2 mM AlocK and spectinomycin and cultured at 37°C and 200 rpm for 5 h. A total of 4 × 10^7^ cells were collected and brought up to 1 mL with LB medium. After centrifugation at 4°C and 6,000 rpm for 3 min, the supernatant was collected and filtered through a 0.22 µm membrane. A 35 µL aliquot of the filtered supernatant was mixed with *E. coli* MG1655 *lacI::cmR* (4.2 × 10^6^ CFUs in 35 µL) and incubated at 37°C for 30 min to allow phage infection. The mixture was then added to 5 mL LB medium and incubated at 37°C for 12 h. The expression of mNG in the cultures was analyzed with flow cytometry. Cultures of *E. coli* K12 WK 6λ *ea47::ampR* and *E. coli* K12 WK 6λ *ea47::mNG* were used as controls.

To assess the independent regulation of different proteins (e.g., mNG and mCherry) by ncAAs (e.g., AlocK and pIF), the plasmid expressing *Mb*PylRS-349F/tRNA^Pyl^ and the Cro-8X mutant was transformed into *E. coli* K12 WK 6λ *ea47::mNG*, and the plasmids expressing *Af*pIFRS/tRNA^Tyr^ and the Cro-14X mutant were transformed into *E. coli* K12 WK 6λ *ea47::mCherry*. For each of the resulting strains, single colonies were inoculated into LB containing antibiotics and cultured at 37°C and 200 rpm for 12 h. The cultures were mixed equally and diluted (1:100) into LB supplemented with or without 2 mM ncAA(s) and antibiotics. After cultivation at 37°C and 200 rpm for 7 h, the culture was collected and processed as described above. The expression of mNG and mCherry was analyzed with flow cytometry.

### Animal studies

C57BL/6J mice (Vital River Laboratory Animal Technology Co., Ltd.) were maintained in the ABSL-2 laboratory at the State Key Laboratory of Virology at Wuhan University.

#### The impact of AlocK on mice

Fifteen 8-week-old female C57BL/6J mice were randomly divided into three groups, and each group was housed in a single cage. After acclimatization for 1 week, the three groups received daily gavage for 11 days with 0.2 mL of PBS, 2 mM AlocK, and 8 mM AlocK solution (prepared in PBS), respectively. The body weight of each mouse was measured daily. On day 12, after a 12 h fast, the mice were anesthetized using an inhalation anesthesia machine (R540, RWD) with 2% isoflurane at an airflow rate of 1.5 L/min, and blood samples were collected for blood tests (Wuhan Servicebio Technology Co., Ltd.). Next, the mice were euthanized with isoflurane, and liver and kidney tissues were harvested for HE staining (Wuhan Servicebio Technology Co., Ltd.).

#### Prophage induction with AlocK *ex vivo*

Six 7-week-old female C57BL/6J mice were randomly divided into two groups, and each group was housed in a single cage. After acclimatization for 1 week, the mice were sacrificed with isoflurane. The colons were collected, with the contents retained to maintain the environment, and injected with 1 × 10^9^ CFUs of the *E. coli* K12 WK 6λ *ea47::ampR* cells carrying the plasmid pUltra-CRSFT-mNG that were suspended in LB with 0 or 8 mM AlocK. After being tied with 5–0 Mersilk suture string (Ethicon), the colons were transferred into Dulbecco’s Modified Eagle’s Medium (low glucose; Cytiva) supplemented with 10% fetal bovine serum (Every Green) and 100 µg/mL spectinomycin, followed by cultivation anaerobically at 37°C for 16 h. The colons were minced and vortexed in 5 mL PBS, followed by passing through a 5 µm filter (Tianjin Jinteng Experiment Equipment Co., Ltd.) and a 0.22 µm filter sequentially to collect λ phage particles. The phage titers were quantified using the double-layer agar method described above.

#### Protein release induced by AlocK *ex vivo*

Six 8-week-old female C57BL/6 J mice were randomly divided into two groups, and each group was housed in a single cage. The mice colons were prepared as described above and injected with 1 × 10^9^ CFUs of the *E. coli* K12 WK 6λ *ea47::ampR* cells carrying the plasmid pUltra-CRSFT-mNG or pUltra-CRSFT that were suspended in LB with 8 mM AlocK. After cultivation anaerobically at 37°C for 16 h, the colon contents were processed as described above. The samples filtered with the 0.22 µm membrane were precipitated with acetone, and the mNG in the resulting pellets was detected by simple western immunoblots.

#### Sustainable protein expression induced by AlocK *ex vivo*

To detect AlocK-induced continuous protein expression, the mice colons from healthy mice were prepared as described above and injected with a mixture of *E. coli* K12 WK 6λ *ea47::mNG* cells carrying the plasmid pUltra-CRSFT (5 × 10^8^ CFUs) and *E. coli* MG1655 *lacI::cmR* cells (5 × 10^8^ CFUs) with or without the supplement of 8 mM AlocK. After cultivation, the colon contents were processed similarly and passed through a 5 µm filter. The resulting samples were serially diluted and plated on LB plates containing ampicillin and chloramphenicol to select colonies of *E. coli* MG1655 *lacI::cmR* infected with λ phages. Phage titers were also determined as described above.

#### Prophage induction by AlocK *in vivo*

Six 8-week-old female C57BL/6J were randomly divided into two groups of three, each housed in a single cage. After acclimatization for 1 week, the mice were subjected to a 2-day fasting period (days 0 to 1) and provided with drinking water containing 2 g/L spectinomycin at day 1. On day 2, after anesthetization with isoflurane, the mice received rectal administration of *E. coli* K12 WK 6λ *ea47::ampR* cells carrying the plasmid pUltra-CRSFT-mNG (1 × 10^9^ CFUs) in PBS with 0 or 8 mM AlocK. Mice were sacrificed the next day, and the colons were collected, followed by processing as described above to quantify the λ phages.

### Statistical analysis

The study data were analyzed statistically using GraphPad Prism 10.1.2 software. Results are presented as mean values with standard deviation. To compare mean differences, an unpaired two-tailed *t*-test was employed. Significance levels are denoted as follows: *P  <  0.05; **P  <  0.005; ***P  <  0.001; ****P  <  0.0001; ns, not significant.

## SUPPLEMENTAL MATERIAL

Supplemental material mbio.03988-24-s0001.pdf Fig. S1 to S17; Tables S1 to S4.

ASM does not own the copyrights to Supplemental Material that may be linked to, or accessed through, an article. The authors have granted ASM a non-exclusive, world-wide license to publish the Supplemental Material files. Please contact the corresponding author directly for reuse.
