# Supplementary material for: Noncanonical amino acids as prophage inducers for protein regulation in bacteria-based delivery systems
Source: mBio. 2025 Mar 14;16(4):e03988-24. doi: 10.1128/mbio.03988-24 (PMC11980383; doi:10.1128/mbio.03988-24)
Supplement: Supplemental material — Fig. S1 to S17; Tables S1 to S4. [file mbio.03988-24-s0001.pdf]

Supplementary Figures

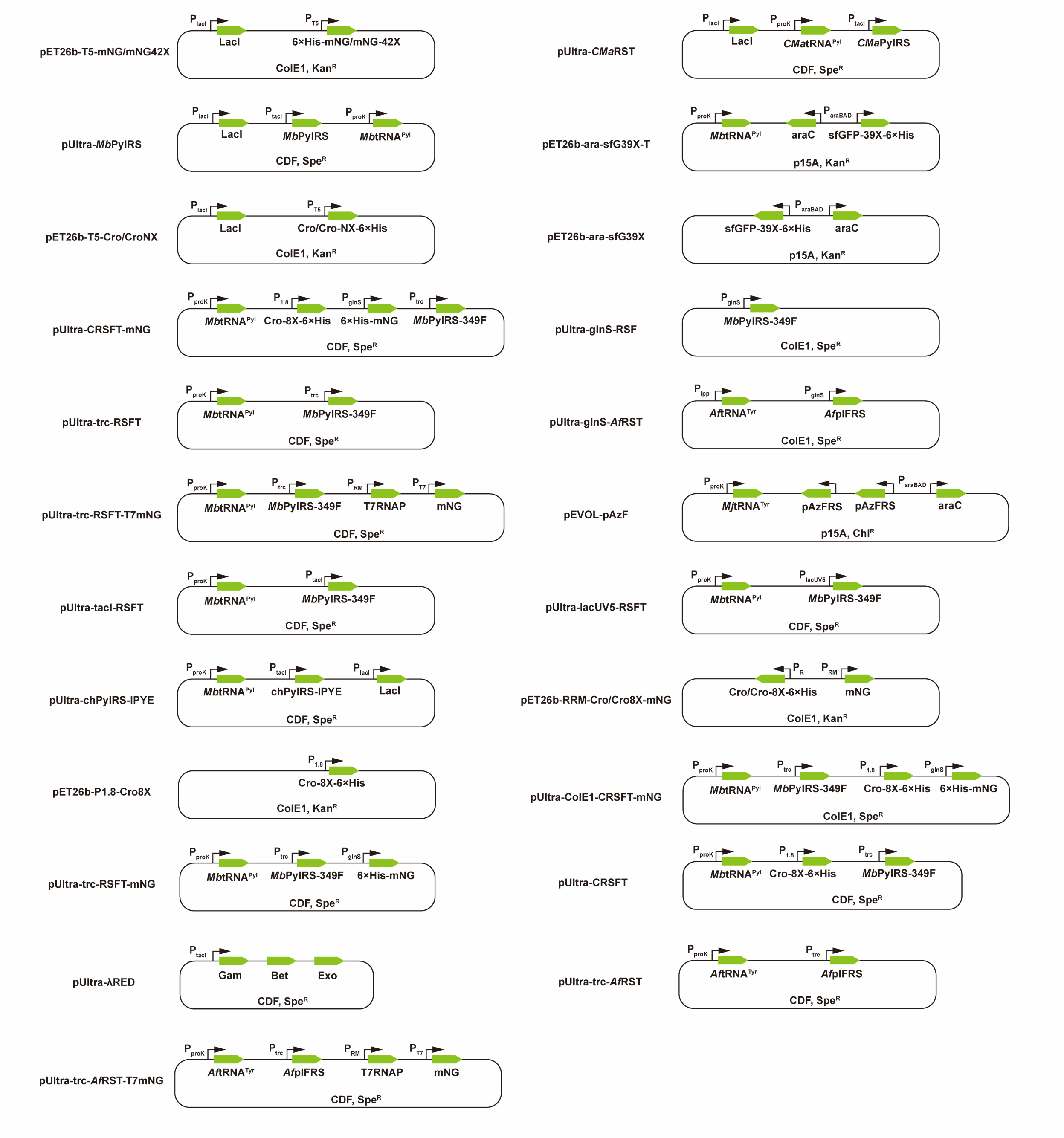

Fig S1. Cartoons of plasmids constructed in this work.

Kan: kanamycin; Spe: spectinomycin; Chl: chloramphenicol.

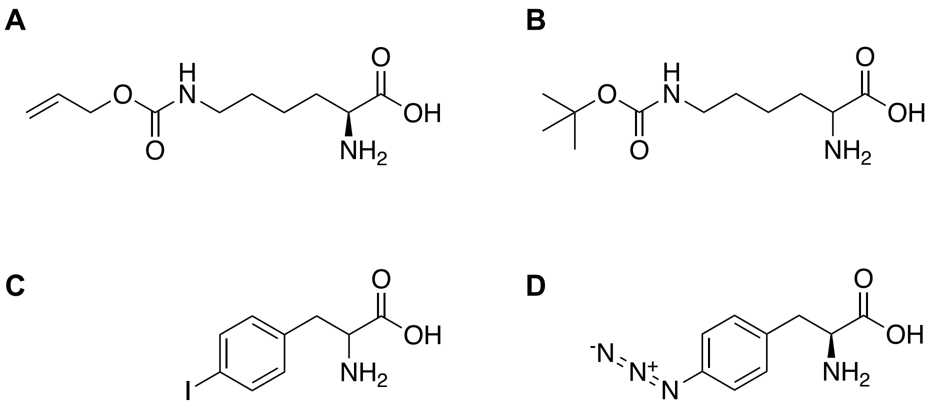

Fig S2. Noncanonical amino acids used in this study. (A) AlocK. (B) BocK. (C) pIF. (D) pAzF.

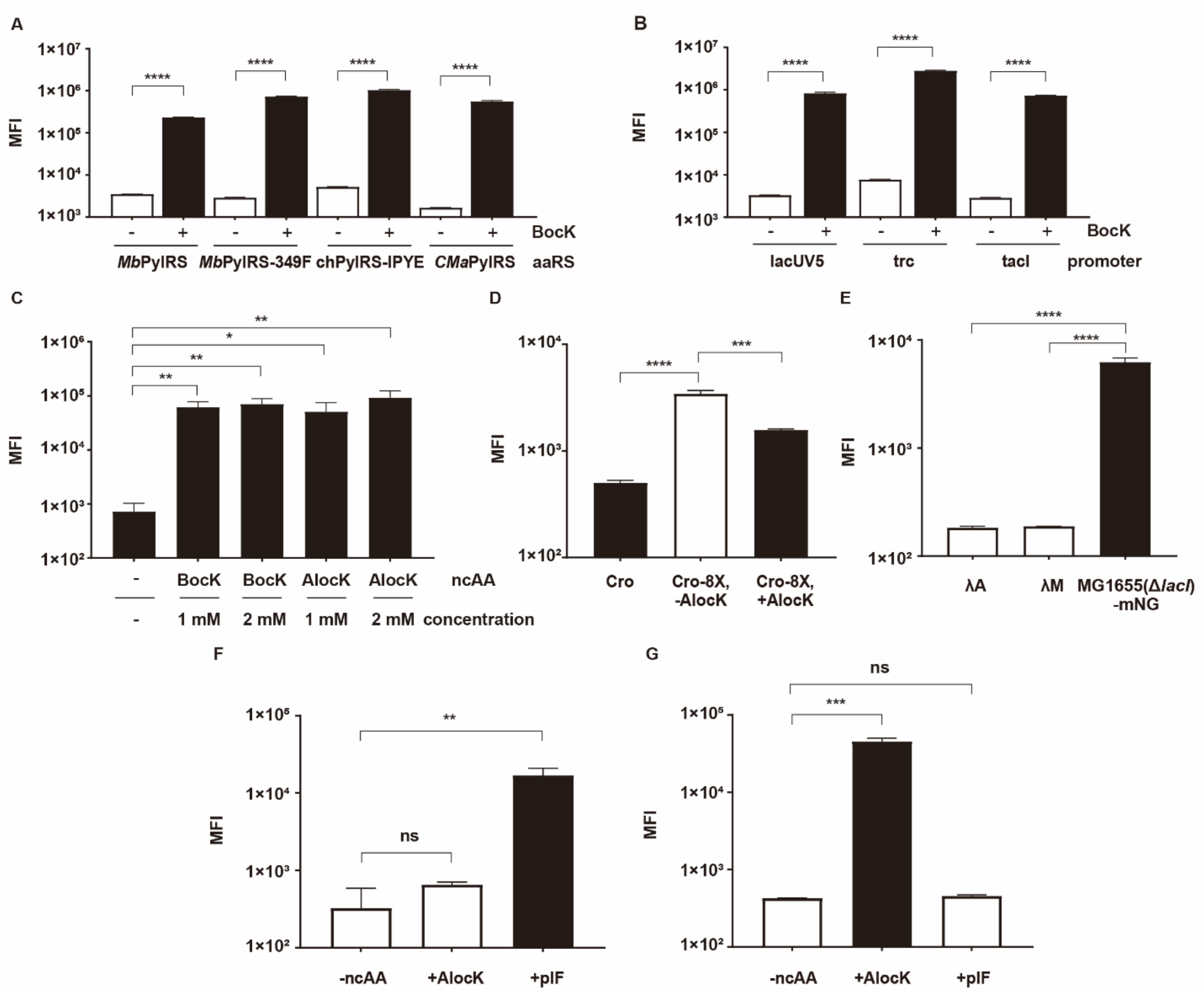

Fig S3. Median fluorescence intensity (MFI) of samples analyzed with flow cytometry in this study. (A) The MFI of samples in Fig 2A. (B) The MFI of samples in Fig 2B. (C) The MFI of samples in Fig 2C. (D) The MFI of samples in Fig 2E. (E) The MFI of samples in Fig 5B. (F) The MFI of samples in Fig 6B. (G) The MFI of samples in Fig 6C. The MFI was analyzed using FlowJo software, and the MFI values of three replicates were presented with the mean and standard deviation (SD). Two-tailed t-tests were performed to compare mean differences; P values indicated were as follows: ****$P < 0.0001$; ***$P < 0.001$; **$P < 0.005$; *$P < 0.05$; ns. not significant.

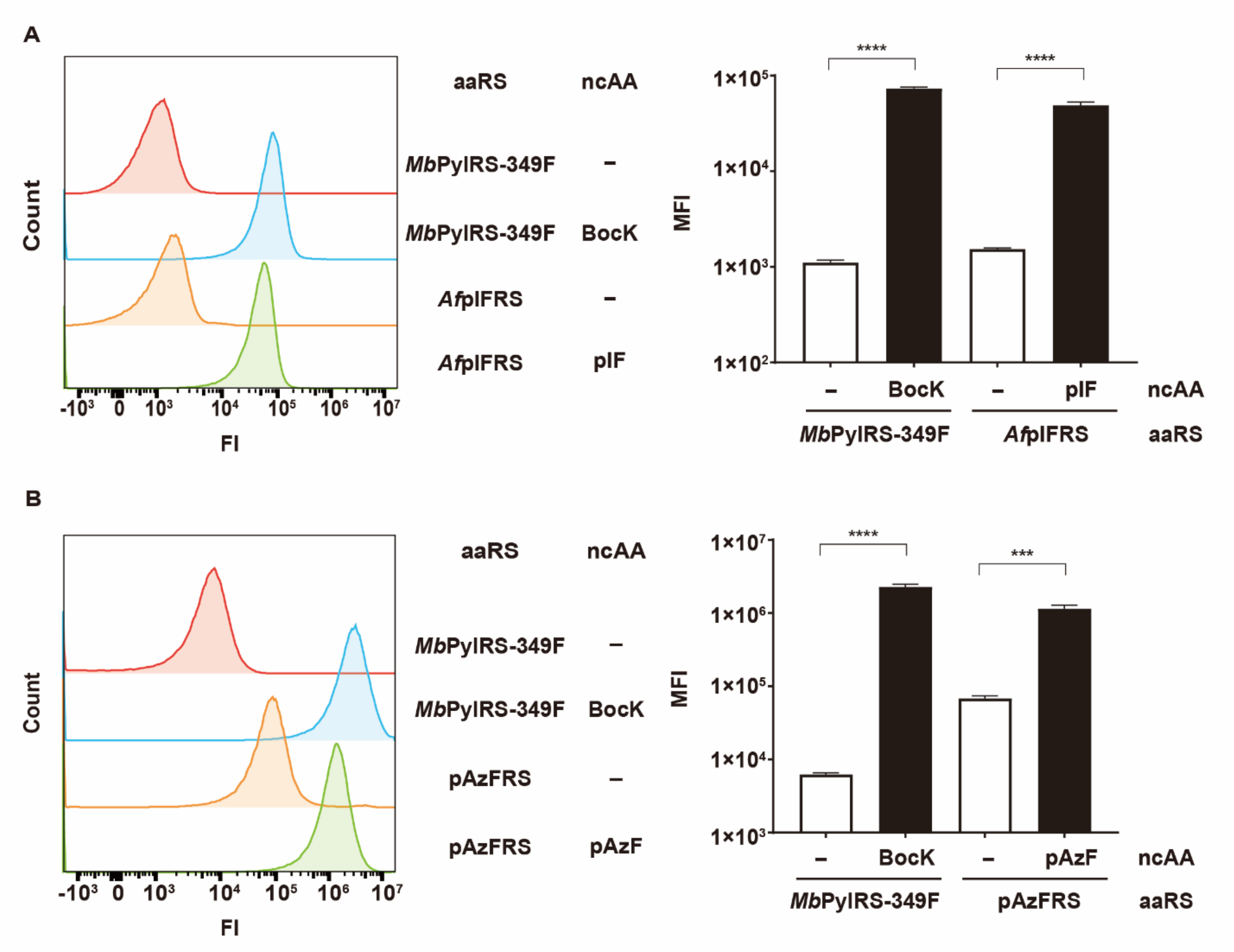

Fig S4. Optimization of the orthogonal translation system. (A) The efficiency of ncAA incorporation by *Mb*PylRS-349F and *Af*pIFRS was tested. Fluorescent signals were detected using flow cytometry with sfGFP-39X as the reporter protein. (B) The efficiency of ncAA incorporation by *Mb*PylRS-349F and pAzFRS was tested. Fluorescent signals were detected using flow cytometry with mNG-42X as the reporter protein. The *Mb*PylRS-349F showed the best incorporation efficiency. The median fluorescence intensity (MFI) values of three replicates were presented in the bar graph (right) with the mean and standard deviation (SD). Two-tailed t-tests were performed to compare mean differences; *P* values indicated were as follows: ****$P < 0.0001$; ***$P < 0.001$.

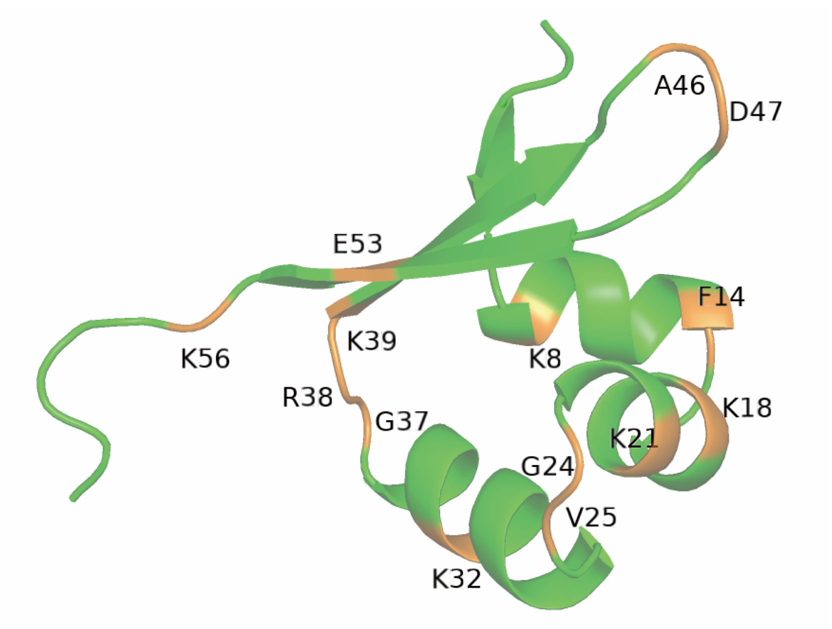

Fig S5. The structure of Cro protein and sites selected for the ncAA incorporation test (PDB = 6CRO). The sites chosen for the ncAA incorporation test are highlighted in orange.

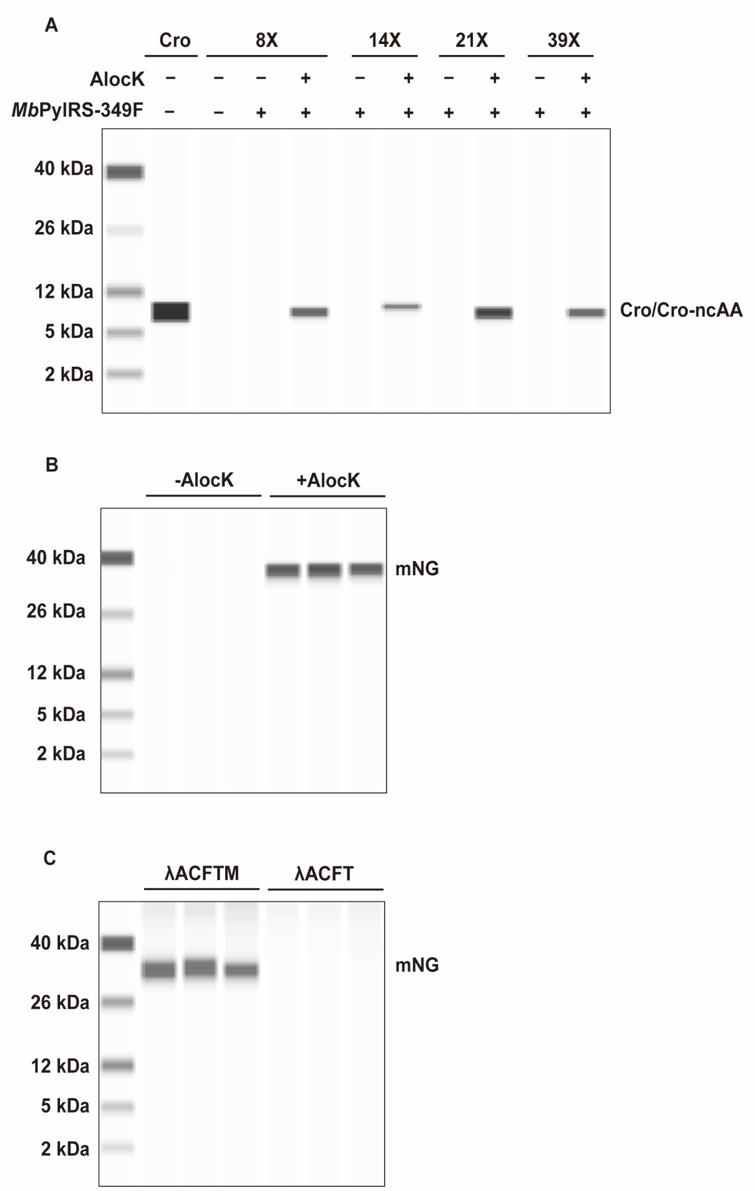

Fig S6. Original simple western immunoblot images of Fig 2D (A), Fig 3E (B), and Fig 4E (C). All protein markers were labeled. mNG: mNeonGreen.

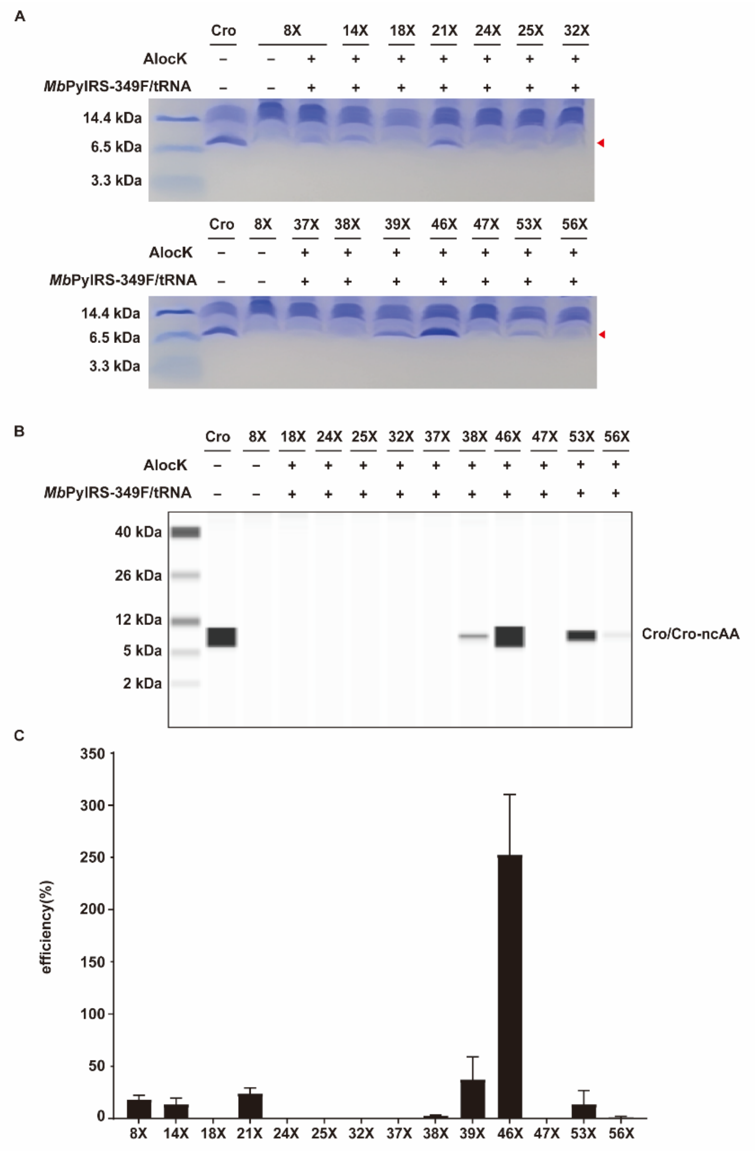

Fig S7. Incorporation of AlocK at different sites of Cro. After the *E. coli* DH10B strain expressing *Mb*PylRS-349F/tRNA^{Pyl} and the variant Cro-NX (a Cro variant with the codon encoding the Nth amino acid mutated to TAG) was induced at 37℃ for 5 hours, whole cell proteins were analyzed by Tricine-SDS-PAGE (A) and simple western immunoblots (B). The strain expressing wild-type Cro or the Cro-8X variant was used as a control. The wild-type Cro or Cro-ncAA variant was marked by the red arrow. (C) The AlocK incorporation efficiency at each tested site (shown in Figs. 2D and S7B) was calculated based on expression levels. The expression level of wild-type Cro was set as 100%.

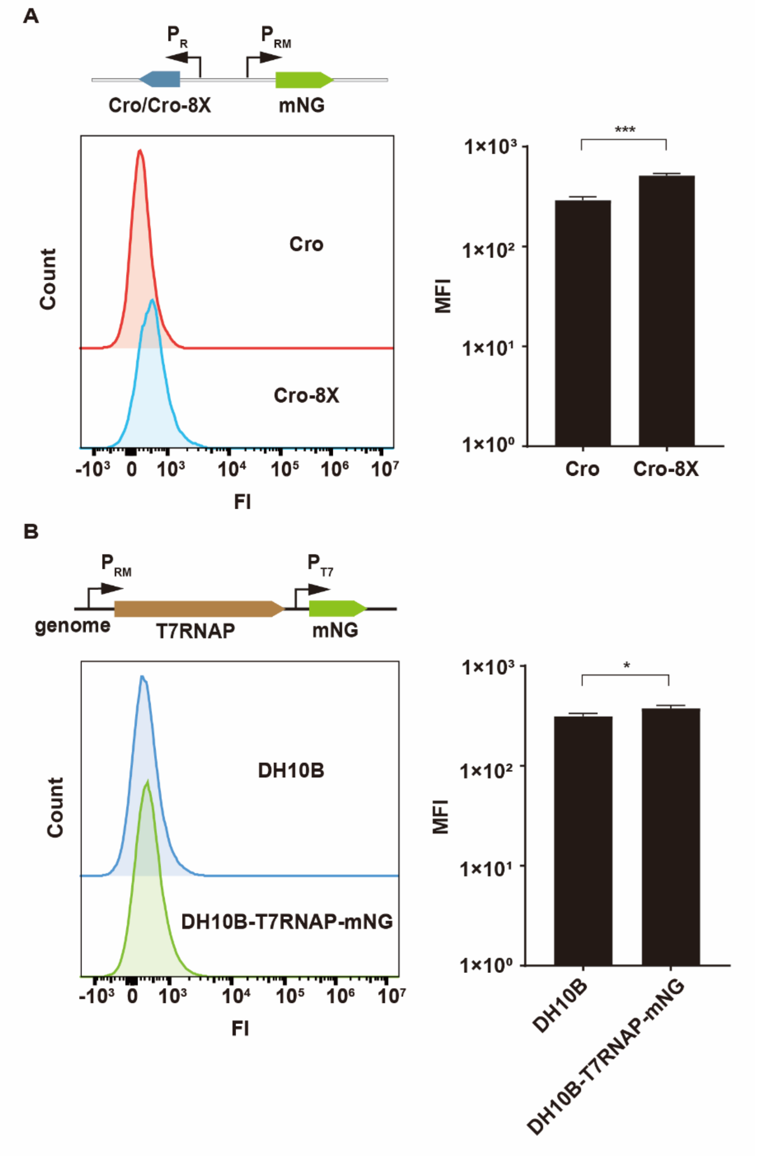

Fig S8. Efforts to set up the function assay for ncAA-containing Cro variants. Flow cytometry analysis was applied to measure the strength of the P_{RM} promoter with a plasmid-based design (A) or by integrating a T7 RNA polymerase-based signal amplification module in the genome of *E. coli* DH10B (B). The median fluorescence intensity (MFI) values of three replicates were presented in the bar graph (right) with the mean and standard deviation (SD). Two-tailed t-tests were performed to compare mean differences; *P* values indicated were as follows: ***$P < 0.001$; *$P < 0.05$.

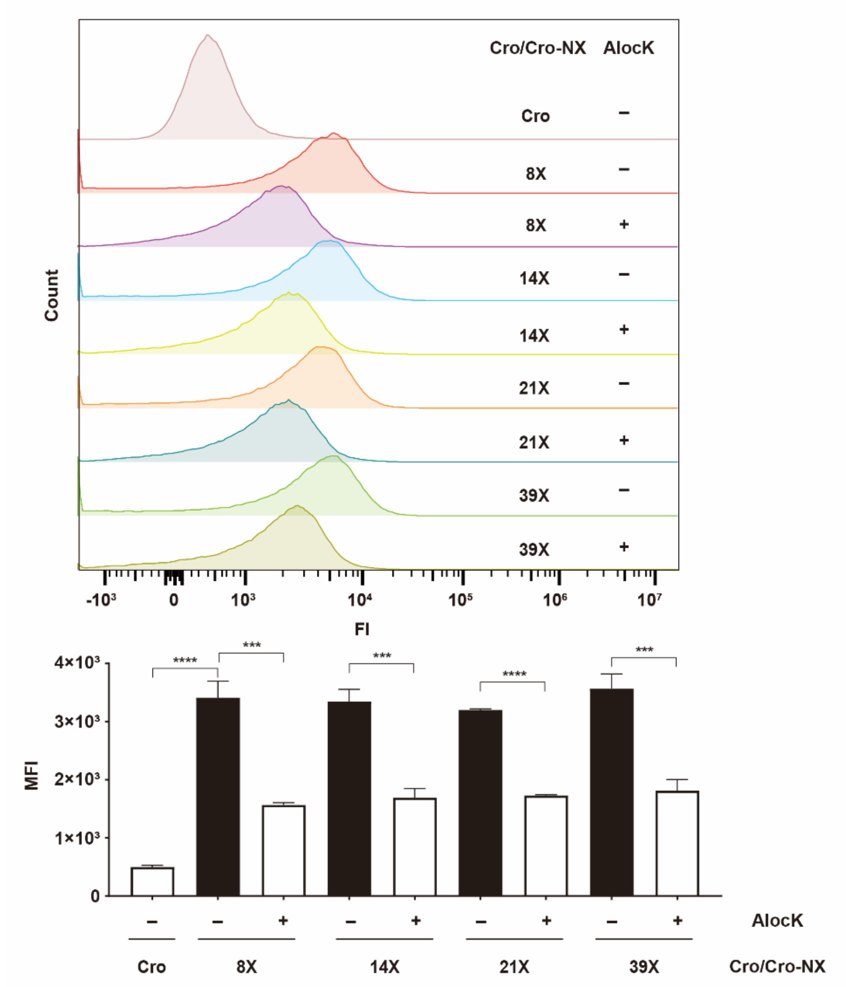

Fig S9. Function assay of Cro variants containing AlocK at a given site. The inhibition of the P_{RM} promoter by Cro variant with AlocK incorporated at positions K8, F14, K21, and K39 was tested (i.e., 8X, 14X, 21X, and 39X). The wild-type Cro was used as the positive control. 2 mM AlocK was used. The fluorescent signals of mNG were detected with flow cytometry. The median fluorescence intensity (MFI) values of three replicates were presented in bar graphs (bottom) with the mean and standard deviation (SD). Two-tailed t-tests were performed to compare mean differences; *P* values indicated were as follows: ****$P < 0.0001$; ***$P < 0.001$.

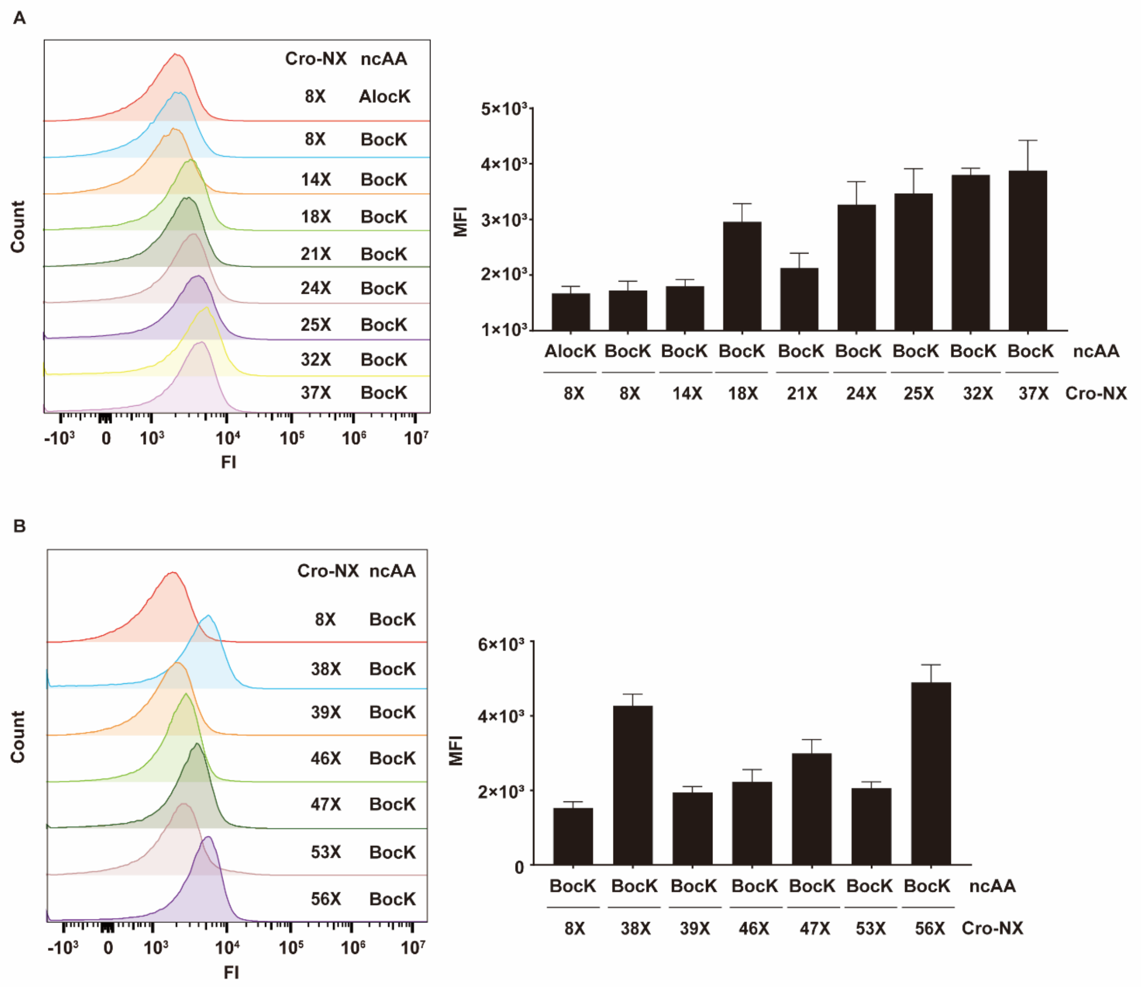

Fig S10. Functional assay of Cro variants containing BocK at a given site. The inhibition effect of the P_{RM} promoter by the Cro variants with BocK incorporated at different sites was tested. 2 mM BocK was used. Cro with AlocK incorporated at position 8 was used as a control. The fluorescent signals of mNG were detected by flow cytometry, and the median fluorescence intensity (MFI) values of three replicates were presented in the bar graph (right) with the mean and standard deviation (SD). Incorporation of BocK at positions 8X and 14X showed the best inhibition effect, comparable to that for AlocK incorporation at position 8X.

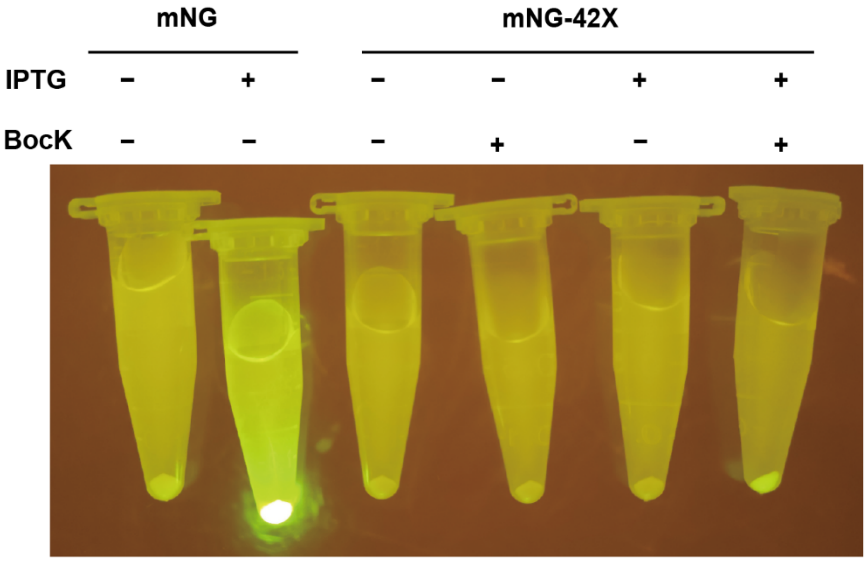

Fig S11. Test of UAG suppression in the λ lysogen strain. The incorporation of the noncanonical amino acid (i.e., BocK) in the λ lysogen strain was analyzed by co-expressing mNG-42X (under the T5 promoter) and *Mb*PylRS/tRNA^{Pyl}. The λ lysogen expressing mNG under the T5 promoter was used as a control. All strains were induced for 5 h at 37°C. Fluorescence of cell pellets excited with blue light (wavelength = 470 nm) was recorded. The strain expressing mNG-42X and *Mb*PylRS/tRNA^{Pyl} did not exhibit fluorescence in the absence of BocK, indicating that natural amino acids were not incorporated at the TAG site (i.e., no UAG suppression naturally occurs in *E.coli* K12 WK 6λ).

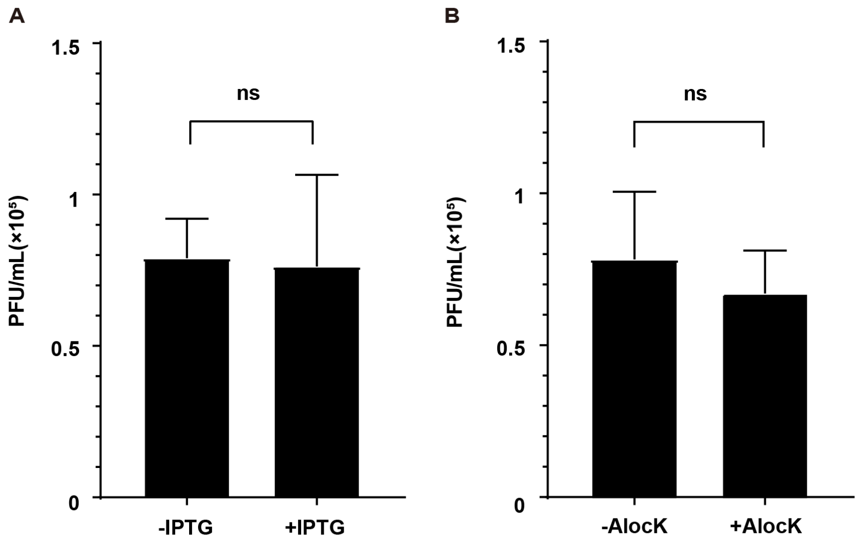

Fig S12. Prophage induction by expression of *Mb*PylRS-349F/tRNA^{Pyl} (A) or supplement of AlocK (B). (A) The amount of λ phages entering the lytic cycle was tested using *E. coli* C600 CR34 as the recipient cells when *E.coli* K12 WK 6λ expressing *Mb*PylRS-349F/tRNA^{Pyl} was treated with or without IPTG. (B) The amount of λ phages entering the lytic cycle was measured when the λ lysogen expressing *Mb*PylRS-349F/tRNA^{Pyl} was treated with or without AlocK. Mean and SD of the number of plaques (PFUs/mL) were presented. Two-tailed t-tests were performed to compare mean differences. ns, not significant. Neither *Mb*PylRS-349F/tRNA^{Pyl} nor AlocK can induce prophage.

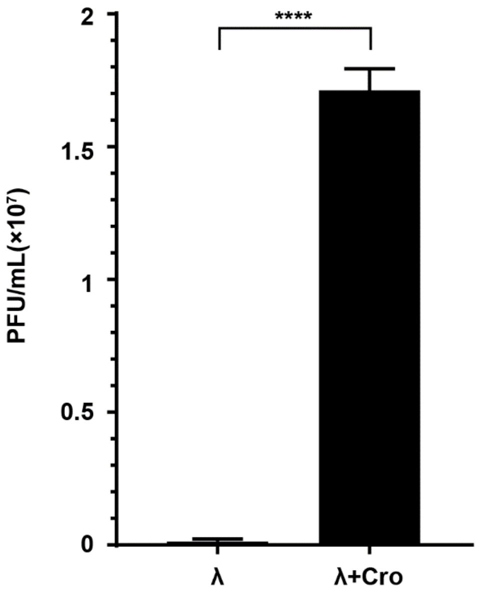

Fig S13. Impact of Cro overexpression in *E.coli* K12 WK 6λ. The *E.coli* K12 WK 6λ strain carrying the recombinant plasmid pET26b-T5-Cro (where Cro was expressed by T5 promoter) was supplemented with 0.01 mM IPTG when sub-cultured (1:100) into fresh LB medium with antibiotics to induce Cro expression until the OD_{600} reached 0.4. The amounts of λ phages entering the lytic cycle were measured, to assay whether overexpression of Cro would lead to prophage activation. "λ" denotes the *E.coli* K12 WK 6λ. "λ+Cro" denotes *E.coli* K12 WK 6λ carrying plasmid pET26b-T5-Cro. Mean and SD of the number of plaques (PFUs/mL) are presented. Two-tailed t-tests were performed to compare mean differences; *P* values indicated were as follows: ****$P < 0.0001$. More λ phages were found to enter the lytic cycle for "λ+Cro", indicating that overexpression of Cro in *E. coli* K12 WK 6λ can induce prophage.

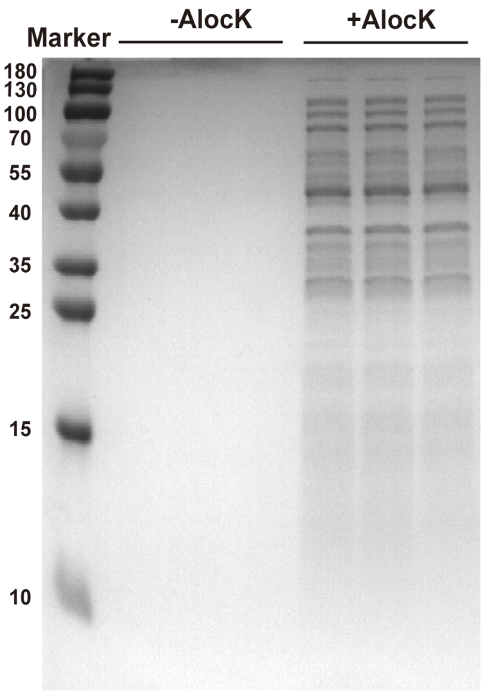

Fig S14. Detection of intracellular proteins released by AlocK-induced bacterial cell lysis with SDS-PAGE. The *E.coli* K12 WK 6λ strain carrying the plasmid pUltra-CRSFT-mNG was supplemented with or without AlocK. The medium was precipitated with acetone, and intracellular proteins released were detected via SDS-PAGE. Three independent biological replicates were performed. The sizes of the protein markers were indicated on the left (kDa).

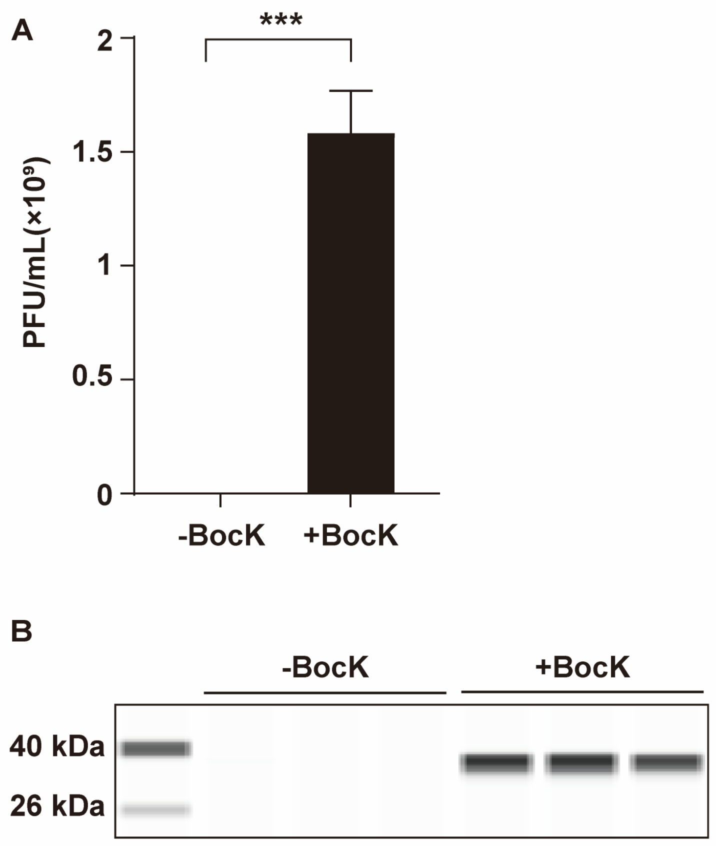

Fig S15. BocK-mediated prophage induction and protein release. The culture of λ lysogen carrying pUltra-CRSFT-mNG was induced with or without BocK for 5 hours, followed by passing through a 0.22 µm filter. The collected samples were subjected to phage titer determination using *E. coli* C600CR34 as the recipient strain (A), and analysis of mNG released with simple western immunoblots using anti-mNeonGreen antibody (B). BocK can induce the prophage, leading to phage-mediated host lysis and release of mNG. The mean and SD of the number of plaques (PFUs/mL) are presented. Two-tailed t-tests were performed to compare mean differences. P values indicated were as follows: ***$P < 0.001$.

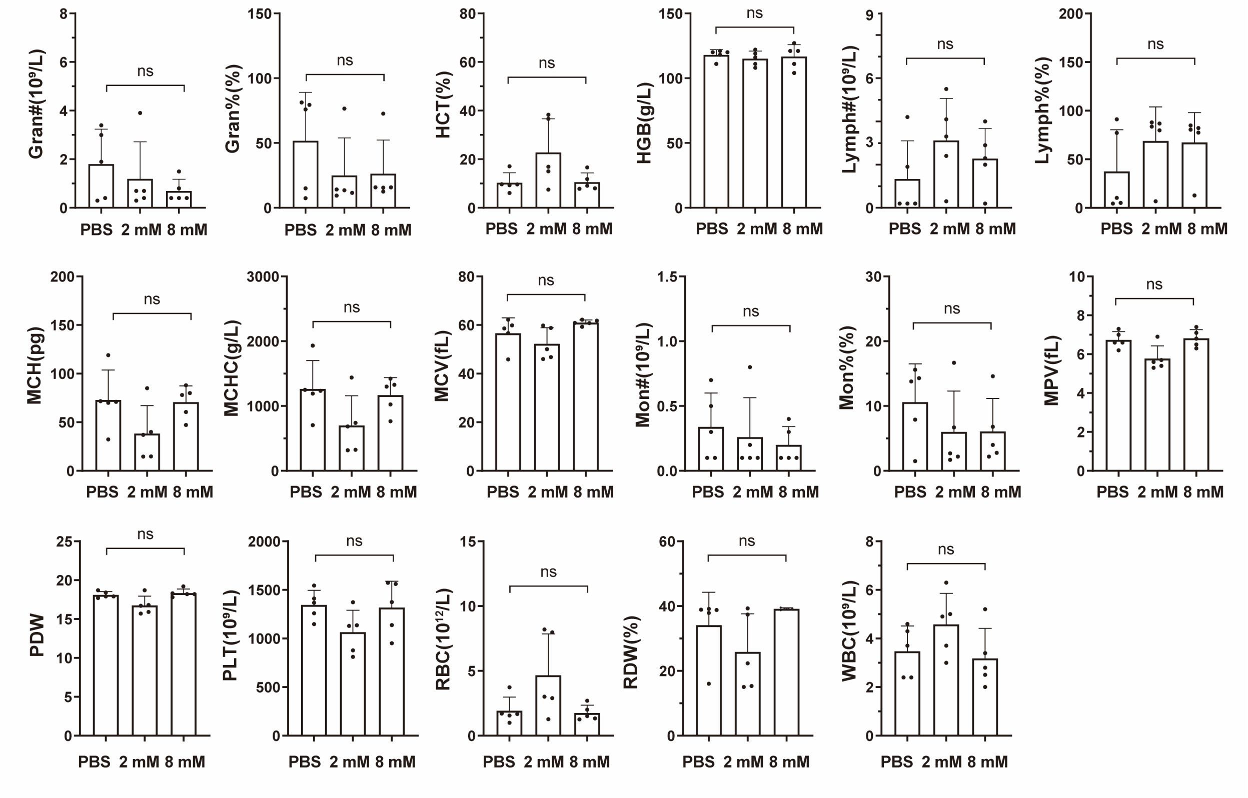

Fig S16. Effect of AlocK treatment on mouse hematology. After the mice received daily gavage with PBS, 2 mM AlocK, or 8 mM AlocK for 11 days, the blood samples were collected for routine hematological analysis. No significant differences in hematological parameters were observed between the groups receiving 8 mM AlocK and PBS. Mean and SD are presented. Two-tailed t-tests were performed to compare mean differences. ns, not significant. Gran, Granulocytes; HCT, Hematocrit; HGB, Hemoglobin; Lymph, Lymphocytes; MCH, Mean cell hemoglobin; MCHC, Mean cell hemoglobin concentration; MCV, Mean cell volume; Mon, Monocytes; MPV, Mean platelet volume; PDW, Platelet Distribution Width; PLT, Platelet count; RBC, red blood cells; RDW, Red blood cell distribution width; WBC, white blood cells.

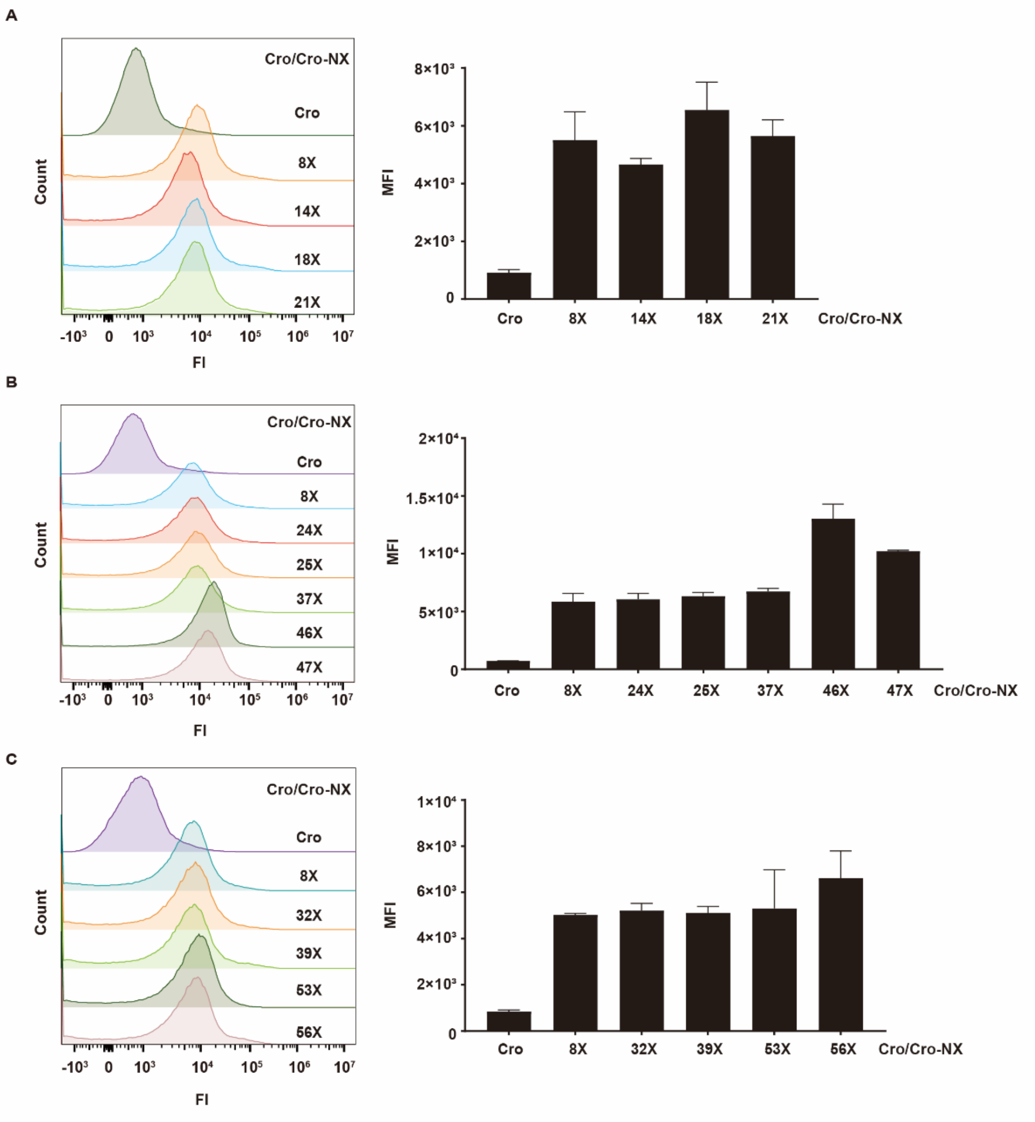

Fig S17. Functional assay of Cro variants containing pIF. The inhibition of the P_{RM} promoter by Cro with pIF incorporated at different positions was tested. 2 mM pIF was used. Wild-type Cro and the Cro variant with pIF incorporated at position 8X were used as controls. The fluorescent signals of mNG were detected by flow cytometry, and the median fluorescence intensity (MFI) values of three replicates were presented in the bar graph (right) with the mean and standard deviation (SD). The incorporation of pIF at position 14 showed the best inhibition effect.

Supplementary Tables

Table S1. Strains used in this work.

Strain Name	Source	Description	Usage
E. coli K12 WK 6λ	CCTCC AB 2013329	-	λ lysogen
E. coli K12 WK 6λ *ea47::ampR*	this work	replace *ea47* with ampicillin resistance gene *ampR*	test the expression of genes integrated in the λ phage genome
E. coli K12 WK 6λ *ea47::mNG*	this work	replace *ea47* with a cassette containing both the *ampR* gene and the *mNG* gene with a T5 promoter	test the expression of genes integrated in the λ phage genome
E. coli K12 WK 6λ ea47::mCherry	this work	replace *ea47* with a cassette containing both the *ampR* gene and the *mCherry* gene with a T5 promoter	test independent regulation of gene expression by different ncAAs
E. coli DH10B	Thermo EC0113	-	test ncAA incorporation efficiency and function of Cro-ncAA
E. coli DH10B-T7RNAP-mNG	this work	insert a cassette containing the *ampR* gene, T7 RNA polymerase gene with a P_{RM} promoter and the *mNG* gene with a T7 promoter	test ncAA incorporation efficiency and function of Cro-ncAA
E. coli C600 CR34	CCTCC AB 2013330	-	quantify the amounts of λ phage as the recipient strain
E. coli MG1655	CCTCC AB 209131	-	model strain used for construct the recipient strain
E. coli MG1655 *lacI::cmR*	This work	replace the *lacI* gene in the genome with chloramphenicol resistance gene *cmR*	recipient strain for testing the expression of genes integrated on the λ phage genome

Table S2. Sequences of proteins and promoters used in this study.

Name	Sequence
**mNeonGreen**	MVSKGEEDNMASLPATHELHIFGSINGVDFDMVGQGTGNPNDGYEELNLKSTKGDLQFSPWILVPHIGYGFHQYLPYPDGMSPFQAAMVDGSGYQVHRTMQFEDGASLTVNYRYTYEGSHIKGEAQVKGTGFPADGPVMTNSLTAADWCRSKKTYPNDKTIISTFKWSYTTGNGKRYRSTARTTYTFAKPMAANYLKNQPMYVFRKTELKHSKTELNFKEWQKAFTDVMGMDELYK*
**mCherry**	MVSKGEEDNMAIIKEFMRFKVHMEGSVNGHEFEIEGEGEGRPYEGTQTAKLKVTKGGPLPFAWDILSPQFMYGSKAYVKHPADIPDYLKLSFPEGFKWERVMNFEDGGVVTVTQDSSLQDGEFIYKVKLRGTNFPSDGPVMQKKTMGWEASSERMYPEDGALKGEIKQRLKLKDGGHYDAEVKTTYKAKKPVQLPGAYNVNIKLDITSHNEDYTIVEQYERAEGRHSTGGMDELYK*
**Cro**	MEQRITLKDYAMRFGQTKTAKDLGVYQSAINKAIHAGRKIFLTINADGSVYAEEVKPFPSNKKTTA*
***Mb*PylRS**	MDKKPLDVLISATGLWMSRTGTLHKIKHHEVSRSKIYIEMACGDHLVVNNSRSCRTARAFRHHKYRKTCKRCRVSDEDINNFLTRSTESKNSVKVRVVSAPKVKKAMPKSVSRAPKPLENSVSAKASTNTSRSVPSPAKSTPNSSVPASAPAPSLTRSQLDRVEALLSPEDKISLNMAKPFRELEPELVTRRKNDFQRLYTNDREDYLGKLERDITKFFVDRGFLEIKSPILIPAEYVERMGINNDTELSKQIFRVDKNLCLRPMLAPTLYNYLRKLDRILPGPIKIFEVGPCYRKESDGKEHLEEFTMVNFCQMGSGCTRENLEALIKEFLDYLEIDFEIVGDSCMVYGDTLDIMHGDLELSSAVVGPVSLDREWGIDKPWIGAGFGLERLLKVMHGFKNIKRASRSESYYNGISTNL*
**sfGFP**	MSKGEELFTGVVPILVELDGDVNGHKFSVRGEGEGDATNGKLTLKFICTTGKLPVPWPTLVTTLTYGVQCFSRYPDHMKRHDFFKSAMPEGYVQERTISFKDDGTYKTRAEVKFEGDTLVNRIELKGIDFKEDGNILGHKLEYNFNSHNVYITADKQKNGIKANFKIRHNVEDGSVQLADHYQQNTPIGDGPVLLPDNHYLSTQSVLSKDPNEKRDHMVLLEFVTAAGITHGMDELYK*
**P1.8 promoter**	TGCTTGACTCGTCGTTATCCTACGTGTATAATTGGC
**lacUV5 promoter**	TTTACACTTTATGCTTCCGGCTCGTATAATG
**proK promoter**	TGTGCTTCTCAAATGCCTGAGGCCAGTTTGCTCAGGCTCTCCCCGTGGAGGTAATAATTGACGATATGATCAGTGCACGGCTAACTAAGCGGCCTGCTGACTTTCTCGCCGATCAAAAGGCATTTTGCTATTAAGGGATTGACGAGGGCGT
**trc promoter**	TTGACAATTAATCATCCGGCTCGTATAATG
**glnS promoter**	TAAAAAACTAACAGTTGTCAGCCTGTCCCGCTTATAAGATCATACGCCGTTATACGTTGTTTACGCTTTGAGGAATCCCAT

Table S3. The construction process of plasmids and strains used in this study.

Plasmid or strain	Construction process
pUltra-CRSFT-mNG	Fragment 1 was PCR-amplified from a synthetic mNG template using primers 113 and 114. Fragment 2 was amplified from pJBEI-6409 (Addgene#47048) using primers 1 and 2. Fragment 3 was amplified from *E. coli* K12 WK 6λ using primers 3 and 4. Fragment 4 was amplified from pUltra-*Mb*PylRS using primers 5 and 6. Fragment 5 was amplified from pUltra-*Mb*PylRS using primers 7 and 8. Fragment 6 was generated by amplifying from Fragment 2 and Fragment 5 using primers 1 and 8. Fragment 7 was generated from Fragment 1 through three rounds of PCR using primer pairs: 9/10, 14/52, and 14/53. Fragment 8 was generated from Fragment 3 via three rounds of PCR using primer pairs: 11/13, 12/55, and 54/56. Fragments 4, 6, 7, and 8 were recombined to form a plasmid 1. Fragment 9 was amplified from plasmid 1 using primers 15 and 16. Fragment 9 was recombined to form the final plasmid.
pET26b-T5-Cro	Fragment 1 was PCR-amplified from pET-26b(+) (Novagen 69862) using primers 17 and 18. Fragment 2 was amplified from *E. coli* K12 WK 6λ using primers 19 and 20. Fragment 3 was generated from Fragment 2 using primers 19 and 21. Fragments 1 and 3 were recombined to form the final plasmid.
pET26b-T5-CroNX	pET26b-T5-Cro was used as the template for PCR amplification of Cro mutants (K8TAG, F14TAG, K18TAG, K21TAG, G24TAG, V25TAG, Y26TAG, K32TAG, G37TAG, R38TAG, K39TAG, A46TAG, D47TAG, E53TAG, and K56TAG.) using specific primer pairs: 22/23, 24/25, 26/27, 28/29, 30/31, 32/33, 34/35, 36/37, 38/39, 40/41, 42/43, 44/45, 46/47, 48/49, 50/51. These fragments were recombined to generate the series of pET26b-T5-CroNX plasmids.
pUltra-trc-RSFT	Fragment 1 was amplified from pJBEI-6409 using primers 58 and 59. Fragment 2 was amplified from pUltra-*Mb*PylRS using primers 60 and 62. Fragment 3 was amplified from pUltra-*Mb*PylRS using primers 57 and 61. These fragments were recombined to form the final plasmid.
pUltra-trc-RSFT-T7mNG	Fragment 1 was amplified from pUltra-trc-RSFT via two rounds of PCR using primer pairs 63/70, and 66/71. Fragment 2 was amplified from a synthetic mNG template using primers 68 and 69. Fragment 3 was amplified from *E. coli* BL21(DE3) via two rounds of PCR using primer pairs 64/67, and 65/67. Fragments 1, 2, and 3 were recombined to form the final plasmid.
pET26b-T5-mNG	Fragment 1 was PCR-amplified from pET-26b (+) via three rounds of PCR using primer pairs using primers 72/73, 75/79, and 76/78. Fragment 2 was amplified using primers 74 and 77. These fragments were recombined to generate the final plasmid.
pET26b-T5-mNG42X	The mNeonGreen gene in pET26b-T5-mNG was mutated at position 42 using primers 80 and 81, generating the final plasmid.
pUltra-tacI-RSFT	Fragment 1 was amplified from pUltra-*Mb*PylRS using primers 83 and 84. Fragment 2 was amplified from pUltra-*Mb*PylRS using primers 82 and 85. These fragments were recombined to form the final plasmid.
pUltra-chPylRS-IPYE	Fragments 1, 2, and 3 were amplified from pUltra-*Mb*PylRS using primer pairs 88/90, 91/93, and 92/94, respectively. These fragments were combined to form Fragment 4. Fragment 5 was amplified from a synthetic *Mm*PylRS template using primers 95 and

	96. Fragment 6 was amplified from pUltra-*Mb*PylRS using primers 86 and 87. Fragments 4, 5, and 6 were recombined to generate the final plasmid.
pUltra-*CMa*RST	Fragment 1 was amplified using primers 99 and 100 from a synthetic *CMa*PylRS template. Fragment 2 was amplified from pUltra-*Mb*PylRS using primers 97 and 98. Fragment 3 was amplified from pUltra-*Mb*PylRS using primers 101 and 102. Fragment 4 was amplified using primers 103 and 104 from a synthetic *CMa*tRNA template. Fragment 5 was generated by amplifying from Fragment 3 and Fragment 4 using primers 105 and 106. Fragments 1, 2, and 5 were recombined to form the final plasmid.
pET26b-ara-sfG39X	Fragment 1 was amplified from pUltra-pAzF using primers 109 and 110. Fragment 2 was amplified from a synthetic sfGFP template using primers 112 and 114, while Fragment 3 was amplified from a synthetic sfGFP template using primers 111 and 113. Fragment 4 was generated by amplifying from Fragment 2 and Fragment 3 using primers 113 and 115. Fragments 5 was amplified from pET26b (+) via three rounds of PCR using primer pairs: 116/117, 116/118, and 116/119. Fragments 1, 4, and 5 were recombined to form the final plasmid.
pET26b-ara-sfG39X-T	Fragment 1 was PCR-amplified from pET26b-ara-sfG39X using primers 120 and 121. Fragment 2 was PCR-amplified from pUltra-*Mb*PylRS using primers 122 and 123. Fragments 1 and 2 were recombined to form the final plasmid.
pUltra-glnS-RSF	Fragment 1 was amplified from pET26b(+) using primers 126 and 127. Fragment 2 was amplified from pUltra-*Mb*PylRS via two rounds of PCR using primer pairs: 124/125, and 134/135. Fragment 3 was amplified from pUltra-*Mb*PylRS using primers 128 and 129. Fragment 4 was amplified from pUltra-*Mb*PylRS using primers 130 and 131. Fragment 5 was generated by amplifying fragments 3 and 4 with primers 132 and 133. Fragments 1, 2, and 5 were recombined to form the final plasmid.
pUltra-glnS-*Af*RST	Fragment 1 was PCR-amplified from pUltra-glnS-RSF using primers 136 and 137. Fragment 2 was amplified from the same template using primers 138 and 139. Fragment 3 was amplified from a synthetic *Af*pIFRS template using primers 144 and 145. Fragment 4 was amplified from a synthetic *Af*tRNA template using primers 142 and 143. Fragment 5 was amplified from the *E. coli* MG1655 genome using primers 140 and 141. Fragment 6 was obtained by amplifying fragments 4 and 5 with primers 140 and 146. Fragments 1, 2, 3, and 6 were recombined to form the final plasmid.
pUltra-lacUV5-RSFT	Fragment 1 was PCR-amplified from pUltra-trc-RSFT using primers 147 and 148. Fragment 2 was generated by amplifying Fragment 1 with primers 149 and 150. Fragment 2 was recombined to form the final plasmid.
pET26b-RRM-Cro-mNG	Fragment 1 was PCR-amplified from pET26b-T5-Cro using primers 151 and 152. Fragment 2 was amplified from pET26b-T5-mNG using primers 153 and 154. Fragment 3 was amplified from pET26b-T5-Cro using primers 155 and 156. Fragment 4 was generated by amplifying Fragment 3 with primers 155 and 157. Fragments 1, 2, and 4 were recombined to create pET26b-RRM-Cro-mNG.
pET26b-RRM-Cro8X-mNG	Fragment 1 was PCR-amplified from pET26b-RRM-Cro-mNG using primers 158 and 159. The resulting fragment was recombined to form the final plasmid.

pET26b-P1.8-Cro8X	Fragment 1 was PCR-amplified from pET26b-T5-Cro8X using primers 160 and 163. Fragment 2 was amplified from the same template using primers 161 and 162. These fragments were recombined to form the final plasmid.
pUltra-trc-RSFT-mNG	Fragment 1 was PCR-amplified from pUltra-trc-RSFT using primers 165 and 166. Fragment 2 was amplified from the same template using primers 164 and 167. Fragment 3 was amplified from pET26b-T5-mNG using primers 168 and 169. Fragments 1, 2, and 3 were recombined to form the final plasmid.
pUltra-ColE1-CRSFT-mNG	Fragment 1 was PCR-amplified from pUltra-CRSFT-mNG using primers 170 and 172. Fragment 2 was amplified from pET26b (+) using primers 171 and 173. The two fragments were recombined to form the final plasmid.
pUltra-CRSFT	Fragment 1 was PCR-amplified from pUltra-CRSFT-mNG using primers 174 and 175. The fragment was recombined to form the final plasmid.
pUltra-λRED	Fragment 1was PCR-amplified from *E. coli* K12 WK 6λ using primers 182 and 184. Fragment 2 was amplified from pUltra-*Mb*PylRS using primers 183 and 185. These fragments were recombined to form the final plasmid.
pUltra-trc-*Af*RST-T7mNG	Fragment 1and fragment 2 were PCR-amplified from pUltra-trc-RSFT-T7mNG using primers 202/203 and 204/205, respectively. Fragment 3 and fragment 4 were amplified frompUltra-glnS-*Af*RST using primers 206/207 and 208/209, respectively. Fragment 5 was generated by amplifying from Fragment 2 and fragment 4 using primers 204 and 210. Fragments 1, 3, and 5 were recombined to form the final plasmid.
pUltra-trc-*Af*RST	Fragment 1 was PCR-amplified from pUltra-trc-RSFT using primers 211 and 212. Fragment 2 was amplified from pUltra-trc-*Af*RST-T7mNG using primers 210 and 213.The two fragments were recombined to form the final plasmid.
DH10B-T7RNAP-mNG	Fragment 1 was PCR-amplified from pUltra-trc-RSFT-T7mNG using primers 179 and 180. Fragment 2 was amplified from pESC-leu2d (empty) (Addgene#20120) using primers 176 and 178. Fragment 3 was generated by amplifying fragments 1 and 2 with primers 177 and 181, and was transformed into *E. coli* DH10B containing the plasmid pUltra-λRED via electroporation. The transformants were selected on LB plate with 25 μg/mL ampicillin, and single colonies were sequenced for verification.
E. coli K12 WK 6λ (*ea47::ampR*)	Fragment 1 was PCR-amplified from pESC-leu2d(empty) using primers 186 and 187. The fragment was transformed into *E. coli* K12 WK 6λ containing the plasmid pUltra-λRED was transformed via electroporation. The transformants were selected on LB plate with 25 μg/mL ampicillin, and single colonies were sequenced for verification.
E. coli K12 WK 6λ (*ea47::mNG*)	Fragment 1 was PCR-amplified from pESC-leu2d(empty) using primers 188 and 189, while Fragment 2 and Fragment 3 were amplified from pET26b-T5-mNeonGreen using primers 190/191 and 192/193, respectively. Fragment 4 was generated by amplifying fragments 1, 2, and 3 with primers 194 and 195. The fragment 4 was transformed into *E. coli* K12 WK 6λ containing the plasmid pUltra-λRED was transformed via electroporation. The transformants were selected on LB plate with 25 μg/mL ampicillin, and single colonies were sequenced for verification.

E. coli K12 WK 6λ (ea47::mCherry)	Fragment 1 was PCR-amplified from pESC-leu2d(empty) using primers 188 and 189. Fragment 2 was amplified from pET26b-T5-mNeonGreen using primers 190 and 191. Fragment 3 was amplified from pUltra-Ptrc-mCherry using primers 192 and 214. Fragment 4 was generated by amplifying fragments 1, 2, and 3 with primers 194 and 195. The fragment 4 was transformed into *E. coli* K12 WK 6λ containing the plasmid pUltra-λRED was transformed via electroporation. The transformants were selected on LB plate with 25 μg/mL ampicillin, and single colonies were sequenced for verification.
MG1655 (*lacI::cmR*)	Fragment 1 was PCR-amplified from *E. coli* MG1655 using primers 196 and 197. Fragment 2 was amplified from pEVOL-pAzF using primers 198 and 199. Fragment 3 was amplified by using primers 200 and 201 as template and primer. Fragment 4 was generated by amplifying fragments 1, 2, and 3 with primers 196 and 201. The fragment 4 was transformed into *E. coli* MG1655 containing the plasmid pUltra-λRED was transformed via electroporation. The transformants were selected on LB plate with 15 μg/mL chloramphenicol, and single colonies were sequenced for verification.

Table S4. Primers used in this study.

Primer	Sequence
Primer 1	AAGAAGATCCTTTGATCGGATCTCGCAAAAAACCCCG
Primer 2	TCCATGTATATCTCCTTCTTAAAAGATCCTGAAATTCCACACATTATACGAGCCGGATGATTAATTGTCA
Primer 3	ATGGAACAACGCATAACCCTGTAGGATTATGC
Primer 4	TCAGTGGTGGTGGTGGTGGTGTGCTGTTGTTTTTTTGTTACTCGGGAAGG
Primer 5	GGTTTTTTGCGAGATCCGATCAAAGGATCTTCTTGAGATCCTTT
Primer 6	CACCGGTTTATTGACTACCGGAAGCAGTGTGACCGTGTGCTTCTCAAATGCCTGAG
Primer 7	TTAAGAAGGAGATATACATGGATAAAAAACCGCTGGACG
Primer 8	CAACTGTTAGTTTTTTAAAGGCCCAGTCTTTCGACT
Primer 9	GCTTTGAGGAATCCCATATGAGAGGATCGCATCACCATCACCATCACGGATCCATGGTGAGCAAGGGCGA
Primer 10	GATCTATCAACAGGAGTCCAAGCTCAGCTAATTAAGCTTGGCTGCAGGTTACTTGTACAGCTCGTCCATG
Primer 11	ACGTGTATAATTGGCTTTCACACAGAATTCATTAAAGAGGAGAAATTAACTATGGAACAACGCATAACCC
Primer 12	AATCATCCCCATAATCCTTGTTAGCCTGCAGGTGCTTGACTCGTCGTTATCCTACGTGTATAATTGGCTT
Primer 13	GCAGCAGCCAACTCAGCTTCCTTTCGGGCTTTGTTAGCAGCCGGATCTCAGTGGTGGTGGTGGTGGTGTG
Primer 14	AAAACTAACAGTTGTCAGCCTGTCCCGCTTATAAGATCATACGCCGTTATACGTTGTTTACGCTTTGAGGAATCCCAT
Primer 15	TGCATGGTGTTTGGCGATACCCTGGA
Primer 16	ATCGCCAAACACCATGCAAGAATCACC
Primer 17	ACAAAGCAAATAAATTTTTTATGATTTCTCGAGAGATCTCGATCCTCTACGCCGGACGC
Primer 18	ACAAAAAAACAACAGCACACCACCACCACCACCACTGAGATCC
Primer 19	TGCTGTTGTTTTTTTGTTACTCGGGAAGG
Primer 20	AGCGGATAACAATTTCACACAGAATTCATTAAAGAGGAGAAATTAACTATGGAACAACGCATAACCCTGA
Primer 21	AAATTTATTTGCTTTGTGAGCGGATAACAATTATAATAGATTCAATTGTGAGCGGATAACAATTTCACAC
Primer 22	AACAACGCATAACCCTGTAGGATTATGCAATGCGCTTTGGG
Primer 23	CAGGGTTATGCGTTGTTCCATAGTT
Primer 24	AAGATTATGCAATGCGCTAGGGGCAAACCAAGACAGCTA
Primer 25	GCGCATTGCATAATCTTTCAGGGTT
Primer 26	ATGCGCTTTGGGCAAACCTAGACAGCTAAAGATCTCGGCGTAT
Primer 27	GGTTTGCCCAAAGCGCATTG

Primer 28	GGCAAACCAAGACAGCTTAGGATCTCGGCGTATATCAAAGCGCG
Primer 29	AGCTGTCTTGGTTTGCCCAAAGCGC
Primer 30	AGACAGCTAAAGATCTCTAGGTATATCAAAGCGCGATCAACA
Primer 31	GAGATCTTTAGCTGTCTTGGTTTGCC
Primer 32	CAGCTAAAGATCTCGGCTAGTATCAAAGCGCGATCAACAAGG
Primer 33	GCCGAGATCTTTAGCTG
Primer 34	CTAAAGATCTCGGCGTATAGCAAAGCGCGATCAACAAGGC
Primer 35	TACGCCGAGATCTTTAGCTGT
Primer 36	ATCAAAGCGCGATCAACTAGGCCATTCATGCAGGCCGAAA
Primer 37	GTTGATCGCGCTTTGATATACGC
Primer 38	ACAAGGCCATTCATGCATAGCGAAAGATTTTTTAACTATAAACGCTGATGGAAG
Primer 39	TGCATGAATGGCCTTGTTGATCG
Primer 40	AGGCCATTCATGCAGGCTAGAAGATTTTTTAACTATAAACGCTGATGGA
Primer 41	GCCTGCATGAATGGCCTTGTT
Primer 42	CCATTCATGCAGGCCGATAGATTTTTTAACTATAAACGCTGATGGAAGCGTTT
Primer 43	TCGGCCTGCATGAATGGCC
Primer 44	TTTTTTAACTATAAACTAGGATGGAAGCGTTTATGCGGAA
Primer 45	GTTTATAGTTAAAAAATCTTTCGGCCTGCATG
Primer 46	TTTTAACTATAAACGCTTAGGGAAGCGTTTATGCGGAAGAGGTA
Primer 47	AGCGTTTATAGTTAAAAAATCTTTCGGCC
Primer 48	ATGGAAGCGTTTATGCGTAGGAGGTAAAGCCCTTCCCGAGTAA
Primer 49	CGCATAAACGCTTCCATCAGC
Primer 50	TTTATGCGGAAGAGGTATAGCCCTTCCCGAGTAACAAAAAAACA
Primer 51	TACCTCTTCCGCATAAACGCTTCCATC
Primer 52	ACCGAGCGTTCTGAACAAATCCAGATGGAGTTCTGAGGTCATTACTGGATCTATCAACAGGAGTCCAAGC
Primer 53	GATTGATGACCGGGAGCTCACAAACATTCTCACCAATAAAAAACGCCCGGCGGCAACCGAGCGTTCTGAA
Primer 54	GCTCCCGGTCATCAATCATCCCCATAATCCTTGTTAGCCTGC
Primer 55	TCAAGACCCGTTTAGAGGCCCCAAGGGGTTATGCTAGTTATTGCTCAGCGGTGGCAGCAGCCAACTCAGC
Primer 56	TAGTCAATAAACCGGTGCTCACATGTTGCGAAGCGGAATTACAAAAAACCCCTCAAGACCCGTTTAGAGG

Primer 57	GATCAAAGGATCTTCTTGAGATCCTTTTTTTCTGC
Primer 58	AAGAAGATCCTTTGATCGGATCTCGCAAAAAACCCCG
Primer 59	GTATATCTCCTTCTTAAAAGATCCTGAAATTGTT
Primer 60	TTAAGAAGGAGATATACATGGATAAAAAACCGCTGGACGT
Primer 61	GTGATTCTTGCATGGTGTTTGGCGATACCCTGGACATCATGCAT
Primer 62	CACCATGCAAGAATCACCGACGATT
Primer 63	GATAATGGTTGCATGTACTAAGGAGGTTGTATGGAACAACGCATAACCCTGTAGCCGCCGCAGTCTCACG
Primer 64	TTGCGGTGATAGATTTAACGTATGACTATGATCACTGTGCATATGAACACGATTAACATCGCTAAGAACG
Primer 65	ACCGCCAGAGGTAAAATAGTCAACACGCACGGTGTTAGATATTTATCCCTTGCGGTGATAGATTTAACGT
Primer 66	ATTTTACCTCTGGCGGTGATAATGGTTGCATGTACTAAGGAGGTTG
Primer 67	TCTAGAGGGGAATTGTTATCCGCTCACAATTCCCCTATAGTGAGTCGTATTATTACGCGAACGCGAAGTC
Primer 68	AACAATTCCCCTCTAGAAATAATTTTGTTTAACTTTAAGAAGGAGATATACATATGGTGAGCAAGGGCGA
Primer 69	ATCTATCAACAGGAGTCCAAGCTCAGCTAATTAAGCTTGGCTGCAGGTTACTTGTACAGCTCGTCCATGC
Primer 70	TGGATTTGTTCAGAACGCTCGGTTGCCGCCGGGCGTTTTTTATTGGTGAGAATGCGCTGCGGACACATAC
Primer 71	GACTCCTGTTGATAGATCCAGTAATGACCTCAGAACTCCATCTGGATTTGTTCAGAACGCTCGG
Primer 72	TTATCCGCTCACAAAGCAAATAAATTTTTTATGATTTCTCGAGAGATCTCGATCCTCTACGCCGGACGCA
Primer 73	GCCAAGCTTGCGGCCGCACTCGAGCACCACCACCACCACCACTGAGATCCG
Primer 74	GAGGAGAAATTAACTATGAGAGGATCGCATCACCATCACCATCACGGATCCATGGTGAGCAAGGGCGAGG
Primer 75	TTCTGTGTGAAATTGTTATCCGCTCACAATTGAATCTATTATAATTGTTATCCGCTCACAAAGCAAATAA
Primer 76	CTCATAGTTAATTTCTCCTCTTTAATGAATTCTGTGTGAAATTGTTATCCG
Primer 77	GGATCTATCAACAGGAGTCCAAGCTCAGCTAATTAAGCTTGGCTGCAGGTTACTTGTACAGCTCGTCCAT
Primer 78	TGGACTCCTGTTGATAGATCCAGTAATGACCTCAGAACTCCATCTGGATTTGTTCAGAACGCTCGGTTGC
Primer 79	TTCAGAACGCTCGGTTGCCGCCGGGCGTTTTTTATTGGTGAGAATCCAAGCTAGCCAAGCTTGCGGCCGC
Primer 80	GCACCGGCAATCCAAATTAGGGTTATGAGGAGTTAAACCTGAAGTCC
Primer 81	ATTTGGATTGCCGGTGCCCTG
Primer 82	ATTCACCACCCTGAATTGACTCTCTTC
Primer 83	AATTCAGGGTGGTGAATGATCAAAGGATCTTCTTGAGATCCTTTTTTTC
Primer 84	GTGATTCTTGCATGGTGTTTGGCGATACCCTGGACATCATGC
Primer 85	CACCATGCAAGAATCACCGACGA

Primer 86	TCTCCACCAACCTGTAAGCGGCCGCGTTTAAACG
Primer 87	TTCGTGGTGTTTAATTTTATGCAGCGT
Primer 88	AAATTAAACACCACGAAATTTCACGTTCGAAAATCTATATCGAAATGGCG
Primer 89	AAATTAAACACCACGAAATTTCACGTTCG
Primer 90	CGAAAGGCACGCGCGGGGCGACAAGAACGGCTATTGT
Primer 91	CCCGCGCGTGCCTTTCGCTATCACAAATACCGCAAAACGTGC
Primer 92	GTGAGTGAGCCGAAAGTTAAAAAAGCGATGCCG
Primer 93	ACTTTCGGCTCACTCACGACGCGAACTTTCACTGA
Primer 94	GCGCTTGCCGGGACAGAGC
Primer 95	CTCTGTCCCGGCAAGCGCCAGCGCGCCGGCCCTGACGAAAAGCC
Primer 96	TTACAGGTTGGTGGAGATGCCATTATAG
Primer 97	AATTCGAAAAGCCTGCTCAACGAG
Primer 98	GTATATTTTACGGTCATGCGGCCGCACCTCCTTTG
Primer 99	ATGACCGTAAAATATACCGACGCGC
Primer 100	TTAGTTGATTTTCGCACCGTTCAGGT
Primer 101	GTGCGAAAATCAACTAAGCGGCCGCGTTTAAACG
Primer 102	GAATGCGGGGCGCATCT
Primer 103	AGATGCGCCCCGCATTCGGGGGACGGTCCGGC
Primer 104	AGCAGGCTTTTCGAATTTGGCGAGAGACCGGGG
Primer 105	GTGCGAAAATCAACTAAGCGGCCG
Primer 106	AGCAGGCTTTTCGAATTTGGCGA
Primer 107	ATGGTGAGCAAGGGCGAGGAGGA
Primer 108	TTACTTGTACAGCTCGTCCATGCCCA
Primer 109	AAGATCAAAGGATCTTCTTTCCATAGGCTCCGCCCCCC
Primer 110	TCTTCTCCTTTGCTCATAGATCTAATTCCTCCTGTTAGCCCAAAAAAAC
Primer 111	GTTTTCCCTATGTAGCATCACCTTCACCC
Primer 112	TGCTACATAGGGAAAACTCACCCTTAAATTTATTTGCACTACT
Primer 113	ATGAGCAAAGGAGAAGAACTTTTCAC
Primer 114	CTAATTAAGCTTGGCTGCAGGTTAGTGGTGGTGGTGGTGGTGGGATCCTTTGTAGAGCTCATCCATGCCA

Primer 115	GATGGAGTTCTGAGGTCATTACTGGATCTATCAACAGGAGTCCAAGCTCAGCTAATTAAGCTTGGCTGCA
Primer 116	GAAGATCCTTTGATCTTTTCTACGGGGTCTG
Primer 117	TTTTCTAAATACATTCAAATATGTATCCGCTCATGAATTAATTCTTAGAAAAACTCATCGAGCATCAAAT
Primer 118	TCGGTTGCCGCCGGGCGTTTTTTATTGGTGAGAATTGTTTATTTTTCTAAATACATTCAAATATGTATCC
Primer 119	GACCTCAGAACTCCATCTGGATTTGTTCAGAACGCTCGGTTGCCGCCGGGC
Primer 120	CACATTCCTCCTTTCAGATTCTCACCAATAAAAAACGCCCGG
Primer 121	AGCTCAGGGTCGAATTTTGTTTATTTTTCTAAATACATTCAAATATGTAT
Primer 122	CTGAAAGGAGGAATGTGCTTCTCAAATGCCTGAGGC
Primer 123	AAATTCGACCCTGAGCTGCTCGA
Primer 124	CCGCGTTCAGCGGCGTTTTTTCTGCTTTTCTTCGCGAATTAATTCCGCTTCGCAAATTCGAAAAGCCTGC
Primer 125	GGCGGAGCCTATGGAAACCGCCGCAGTCTCACGC
Primer 126	CGTATGATCTTATAAGCGGGACAGGCTGACAACTGTTAGTTTTTATTGAGATCCTTTTTTTCTGCGCGT
Primer 127	TTTCCATAGGCTCCGCCCCCCTGA
Primer 128	CCTGATAAGCGTAGCGCATCAGGCAATTTAGCGTTTGAAACTGCAGTTATTACAGATTGGTTGAGATGCC
Primer 129	TTCTTGCATGGTGTTTGGCGATACCCTGGACA
Primer 130	AAACACCATGCAAGAATCACCGACGA
Primer 131	CTTATAAGATCATACGCCGTTATACGTTGTTTACGCTTTGAGGAATCCCATATGGATAAAAAACCGCTGG
Primer 132	CTTATAAGATCATACGCCGTTATACGTTGTT
Primer 133	CTTATCCGGCCTACAAAAGCACGCAAACTCAATATATTGCAGAGATCATGTAGGCCTGATAAGCGTAGCG
Primer 134	GTAGGCCGGATAAGGCGTTCACGCCGCATCCGGCAAGAAACAGCAAACAATCCAAAACGCCGCGTTCAGC
Primer 135	GGCGGAGCCTATGGAAACCGC
Primer 136	AATTCGAAAAGCCTGCTCAACG
Primer 137	GGATTCCTCAAAGCGTAAACAACG
Primer 138	TGGGTGTGTCCGTGTAACTGCAGTTTCAAACGCTAAATTGC
Primer 139	AATTCACAAAAGCAATATGCGAAGCGGAATTAATTCGCGAA
Primer 140	TATTGCTTTTGTGAATTAATTTGTATATCG
Primer 141	TATTAATACCCTCTAGATTGAGTTAATCTC
Primer 142	AGCAGGCTTTTCGAATTTGGTCCCGCCCCCAG
Primer 143	TCTAGAGGGTATTAATACCCGCCCTAGCTCAGAGG

Primer 144	TACGCTTTGAGGAATCCCATATGGACATTACCGAAAAACTGCG
Primer 145	TTACACGGACACACCCAGGCG
Primer 146	AGCAGGCTTTTCGAATTTGGTC
Primer 147	AAGCCTGGGGTGCCTAATGAGTGAGCTAACTCACATTAATTGCGTTGCGCGATCAAAGGATCTTCTTGAG
Primer 148	GAATTATGAGCGGATAATAATTTCACACAGGAAACAGCTAGGAGGTCAGATTATGGATAAAAAACCGCTG
Primer 149	TTATCCGCTCATAATTCCACACATTATACGAGCCGGAAGCATAAAGTGTAAAGCCTGGGGTGCCTAA
Primer 150	GAATTATGAGCGGATAATAATTTCACACAG
Primer 151	TCTTGAGGGGTTTTTTGTGCTGCAAAACGTCTGCGACC
Primer 152	TTTTTATTGGTGAGAATCTGAAAGGAGGAACTATATCCG
Primer 153	ATTCTCACCAATAAAAAACGCCCGGC
Primer 154	ATAGTCAACACGCACGGTGTTAGATATTTATCCCTTGCGGTGATAGATTTAACGTATGGTGAGCAAGGGC
Primer 155	CAAAAAACCCCTCAAGACCCGT
Primer 156	ACTATTTTACCTCTGGCGGTGATAATGGTTGCATGTACTAAGGAGGTTGTATGGAACAACGCATAACCCT
Primer 157	CCGTGCGTGTTGACTATTTTACCTCTGGCGGTGAT
Primer 158	CATAACCCTGTAGGATTATGCAATGCGCTTTGGGC
Primer 159	ATTGCATAATCCTACAGGGTTATGCGTTGTTCC
Primer 160	GCGCAACGCAATTAATGTAAGTTAGCT
Primer 161	CATTAATTGCGTTGCGCCGGGATCTCGACGCTCTCC
Primer 162	ATGAATTCTGTGTGAAAGCCAATTATACACGTAGGATAACGACGAGTCAAGCATTTCTCGAGAGATCTCG
Primer 163	TTTCACACAGAATTCATTAAAGAGGAGAAATTAACTATGGAACAACGCATAACCCT
Primer 164	TCCGGCTCGTATAATGTGTGGAATTTCAGGATCTTTTAAGAAGGAGATATACATGGATAAAAAACCGCTG
Primer 165	ACATTATACGAGCCGGATGATTAATTGTCAACAGCTCATTTCAGAATATTTG
Primer 166	TTTTTATTGGTGAGAATGTTTGTGAGCTCCCGGTC
Primer 167	GTATAACGGCGTATGATCTTATAAGCGGGACAGGCTGACAACTGTTAGTTTTTAAAGGCCCAGTCTTTC
Primer 168	ATTCTCACCAATAAAAAACGCCCG
Primer 169	CATACGCCGTTATACGTTGTTTACGCTTTGAGGAATCCCATATGAGAGGATCGCATCACCATCACCATCA
Primer 170	GGATCTCGCAAAAAACCCCG
Primer 171	GGTTTTTTGCGAGATCCTTGAGATCCTTTTTTTCTGCGCGTA
Primer 172	CCGCCGCAGTCTCACG

Primer 173	GCGTGAGACTGCGGCGGTTTCCATAGGCTCCGCCCCC
Primer 174	ACCGGGAGCTCACAAACAAGGCCCAGTCTTTCGACTGAGCCTT
Primer 175	GTTTGTGAGCTCCCGGTCATCAATCATCC
Primer 176	TTGGTCTGGTGTCAAAAATAAGAAGATCCTTTGATCTTTTCTACGGGGTCTG
Primer 177	AGCTGAGCGCCGGTCGCTACCATTACCAGTTGGTCTGGTGTCAAAAATAAGAAGA
Primer 178	CGCGGAACCCCTATTTGTTTATTTTTCTAAAT
Primer 179	CAAATAGGGGTTCCGCGATTCTCACCAATAAAAAACGCCCGGC
Primer 180	GCAGACATGGCCTGCCCGGTTATTACTACAGGGTTATGCGTTGTTCCAT
Primer 181	ATGGATTTCCTTACGCGAAATACGGGCAGACATGGCCTGCCCGGTTATTA
Primer 182	TCATCGCCATTGCTCCCCAAATACAAA
Primer 183	GGGAGCAATGGCGATGAGCGGCCGCGTTTAAACGGT
Primer 184	ATGGATATTAATACTGAAACTGAGATCAAGCAAAAGCA
Primer 185	TCAGTATTAATATCCATGCGGCCGCACCTCCTTTG
Primer 186	TCTAAAATATATCAGCATCTAGCATGCAACCTATCAAAATGGAGAGTTTTATGAGTATTCAACATTTCC
Primer 187	GGTTTATTTGGCGATTATTATCTTCAGGAGAATAATGGAAGTTCTATGACTTACCAATGCTTAATCAGTG
Primer 188	GAAGATCCTTTGATCTTTTCTACGGGGTCTGACG
Primer 189	TCTAAAATATATCAGCATCTAGCATGCAACCTATCAAAATGGAGAGTTTTATGAGTATTCAACATTTCCG
Primer 190	GATCAAAGGATCTTCTCATAAAAAATTTATTTGCTTTGTGAGCG
Primer 191	GCCCTTGCTCACCATAGTTAATTTCTCCTCTTTAATGAATTCTGTGTG
Primer 192	GGTTTATTTGGCGATTATTATCTTCAGGAGAATAATGGAAGTTCTATGACTTACTTGTACAGCTCGTCCATG
Primer 193	ATGGTGAGCAAGGGCGAGGAGGATAAC
Primer 194	TCTAAAATATATCAGCATCTAGCATGCAAC
Primer 195	GGTTTATTTGGCGATTATTATCTTCAGG
Primer 196	AAGCCTGGGGTGCCTAATGAGTGAGCTAAC
Primer 197	CGCAATTAATGTGAGTTAGCTCACTCATTAG
Primer 198	ACTCACATTAATTGCGTTGCGCTTACGCCCCGCCCTGCCAC
Primer 199	TCAATTCAGGGTGGTGAATGTGAAACCAGTAACGTGATCGGCACGTAAGAGGTTCCAAC
Primer 200	ATTCACCACCCTGAATTGACTCTC
Primer 201	GCGGTATGGCATGATAGCGCCCGGAAGAGAGTCAATTCAGGGTG

Primer 202	AATTCGAAAAGCCTGCTCAACGAGC
Primer 203	GTATATCTCCTTCTTAAAAGATCCTGAAATTG
Primer 204	GCGGCCGCGTTTAAACGGTCTC
Primer 205	GAATGCGGGGCGCATCTTACTGCGCAGATAC
Primer 206	CTTTTAAGAAGGAGATATACATGGACATTACCGAAAAACTGCGTC
Primer 207	TTTAAACGCGGCCGCTTACACGGACACACCCAGGCGTTTACGAGC
Primer 208	TAAGATGCGCCCCGCATTCCCCGCCCTAGCTCAGAGGTAGAG
Primer 209	TTGAGCAGGCTTTTCGAATTTGGTCCCGCCCCCAGGATTTG
Primer 210	TTGAGCAGGCTTTTCGAATTTGGTC
Primer 211	CGAAAAGCCTGCTCAACGAGCAGGCTTTTTTGCATGCTCGAGCAGC
Primer 212	CCATGTATATCTCCTTCTTAAAAGATCCTGAAATTGTTATC
Primer 213	GAAGGAGATATACATGGACATTACCGAAAAACTGCGTC
Primer 214	ATGGTGAGCAAGGGCGAGGAGGATAACATG
